# Fruit Development in *Ficus carica* L.: Morphological and Genetic Approaches to Fig Buds for an Evolution From Monoecy Toward Dioecy

**DOI:** 10.3389/fpls.2020.01208

**Published:** 2020-08-21

**Authors:** Ilaria Marcotuli, Andrea Mazzeo, Pasqualina Colasuonno, Roberto Terzano, Domenica Nigro, Carlo Porfido, Annalisa Tarantino, Riccardo Aiese Cigliano, Walter Sanseverino, Agata Gadaleta, Giuseppe Ferrara

**Affiliations:** ^1^Department of Environmental and Territorial Sciences, University of Bari “Aldo Moro”, Bari, Italy; ^2^Department of Soil, Plant and Food Sciences, University of Bari “Aldo Moro”, Bari, Italy; ^3^Department of Agriculture, Food and Environmental Sciences, University of Foggia, Foggia, Italy; ^4^Sequentia Biotech SL, Barcelona, Spain

**Keywords:** *Ficus carica* L., breba, main crop, RNA seq, transcriptome analysis, 3D X-ray tomography, anatomical analysis

## Abstract

The mechanism behind the bud evolution towards breba or main crop in *Ficus carica L*. is uncertain. Anatomical and genetic studies may put a light on the possible similarities/differences between the two types of fruits. For this reason, we collected complimentary data from anatomical, X-ray imaging, and genetic techniques. The RNA seq together with structural genome annotation allowed the prediction of 34,629 known genes and 938 novel protein-coding genes. Transcriptome analysis of genes during bud differentiation revealed differentially expressed genes in two fig varieties (Dottato and Petrelli) and in breba and main crop. We chose Dottato and Petrelli because the first variety does not require pollination to set main crop and the latter does; moreover, Petrelli yields many brebas whereas Dottato few. Of the 1,615 and 1,904 loci expressed in Dottato and Petrelli, specifically in breba or main crop, respectively, only 256 genes appeared to be transcripts in both varieties. The buds of the two fig varieties were observed under optical microscope and using 3D X-ray tomography, highlighting differences mainly related to the stage of development. The X-ray images of buds showed a great structural similarity between breba and main crop during the initial stages of development. Analysis at the microscope indicated that inflorescence differentiation of breba was split in two seasons whereas that of main crop started at the end of winter of season 2 and was completed within 2 to 3 months. The higher expression of floral homeotic protein AGAMOUS in breba with respect to main crop, since this protein is required for normal development of stamens and carpels in the flower, may indicate an original role of these fruits for staminate flowers production for pollination of the main crop, as profichi in the caprifig. Several genes related to auxin (auxin efflux carrier, auxin response factor, auxin binding protein, auxin responsive protein) and to GA synthesis (GA20ox) were highly expressed in brebas with respect to main crop for the development of this parthenocarpic fruit.

## Introduction

*Ficus carica* L. is one of the ≅700 species of the *Ficus* genus in the *Moraceae* family (Datwyler and Weiblen, 2004; Flaishman et al., 2008). Fig was a popular fruit in the diet of Roman people, as supported by many relics found in cities such as Pompeii, Oplontis, and in different areas of the Roman Empire, from Africa to Europe. Cato, Pliny the Elder, and Columella described horticultural practices (for example, tillage and fertilization) and varieties (African, Winter, Tiburtine, Pompeian, Herculanean, Saguntine), but they did not specifically describe the breba or first crop (generally ripening in May-June) and the main crop (ripening in July-September). The fig fruit is a composite type of fruit and consists of a receptacle tissue enclosing hundreds of individual pedicellate pistillate flowers developing into drupelets. The fertilized drupelets contain small formed seeds with respect to the empty drupelets of the parthenocarpic (persistent) fruits. The composite fruit developing from this inflorescence is called syconium. The first crop is called breba, ripening in May-June in the Northern Hemisphere, with fruits generally larger than the main crop and persistent. The second crop is the main crop (forniti) with a possible third crop harvested in autumn; the second crop may or may not be pollinated (Storey, 1977; Stover et al., 2007; Flaishman et al., 2008; Ferrara et al., 2016). Botanically, the fig is a gynodioecious species, and female trees (*Ficus carica sativa* L.) need to be pollinated, or “caprificated”, by the male trees (*Ficus carica caprificus* L.) to set the main crop. In the process of caprification, wasps (*Blastophaga psenes* L.) passively carry pollen from male flowers, located in the inedible profichi fruits borne on caprifig trees (hermaphroditic, with male and short-styled female flowers in the profichi fruits), to the long-styled female flowers of the edible figs (in the main crop). Apart from naturally occurring pollination where caprifig trees are present, caprification by growers has been practiced in the Mediterranean area for centuries, as documented by Aristotle, Theophrastus, and Pliny the Elder (Vallese, 1909; Siniscalchi, 1911; Condit, 1955). In brief, growers placed some profichi fruits, containing pollen and pollinators, on female trees to facilitate pollination of female flowers in edible figs. Collecting profichi and putting them in trees is laborious and costly, so this process has fallen out of favor and varieties have been selected that do not require caprification. The eradication of caprifigs has led to a simplification/erosion of fig biodiversity, in particular of varieties requiring pollination.

The reproductive biology of fig is very complex, since there are fig varieties which need pollination and varieties that do not. As a consequence, edible fig varieties have been grouped in three main groups: 1) the Common type, with trees developing fruits parthenocarpically (persistent), either brebas or main crop; 2) the Smyrna type, non-parthenocarpic, which requires pollination to bear the main crop and do not usually produce brebas; 3) the San Pedro type, bearing brebas parthenocarpically and the main crop generally after caprification or even parthenocarpically (Storey, 1977; Ferrara et al., 2016). In some varieties, the main crop could be divided in two sub-groups, the (summer) main crop (ripening in July-September) and the late main crop ripening in autumn and borne on the trees up to December. There are varieties which produce only the main crop ripening very late (cv. Natalino) and eaten almost at Christmas time.

**Figure 1:**
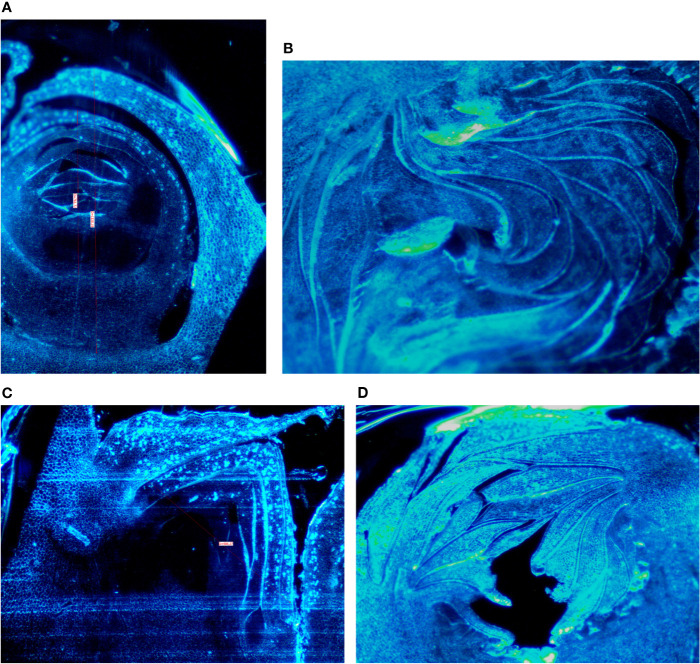
Section of fruit buds of Petrelli in distal nodes **(A)** and basal ones **(B)**. Section of fruit buds of Dottato in distal nodes **(C)** and basal ones **(D)**.

The male fig (caprifig) can have three fruits similarly to the female fig: 1) the profichi in spring-beginning summer; 2) the mammoni in summer-autumn; and 3) the mamme during autumn-winter. Either the presence or the amount of the three types of fruits may vary consistently among varieties of caprifig. The male flowers for the caprification of the main crop in the edible fig are present in the profichi of the caprifig (**Figure S1**). Female flowers are either short-styled or long-styled, with the first type located in the fruits of the caprifig and the second type in the fruits of the edible fig (**Figure S2**). All these flowers are inserted on a receptacle forming the syconium, which is palatable in the edible fig, and generally non-palatable in the caprifig (caprifig means fig for the goats and not for human beings). Short-styled flowers are used by the wasps for oviposition in the ovary whereas the long-styled ones are not efficient for the wasps because the style is longer than the ovipositor.

All the fig buds are developed on the same shoot (**Figure S3**), i.e., fruit bud, vegetative bud and mixed bud. In particular, the apical/lateral bud is a mixed bud which in spring originates a shoot carrying axillary fruits and/or buds developing in the current and/or successive season all the crops. In the common fig, the basal portion of the shoot is generally occupied by the main crop and the distal by the brebas (sometimes with the late ripening main crop in the middle), but some overlapping situations may occur. In the caprifig in the basal portion, there are the mammoni, then the mamme and finally the profichi in the distal portion, when all these types of fruits are present.

This “difference” of crops lead to the distinction of the varieties in uniferous (only one crop, main crop), biferous (two crops, breba, and main), and even triferous (breba, summer, and late main crop) (Ferrara et al., 2017; Marcotuli et al., 2019). *It is unclear why some fruit buds on the shoot may develop in summer for the main crop, or in autumn-winter for the late main crop or even in the following year (breba) since they originated from the same mixed bud bursting in the current season*.

The aim of the present work was to study the buds of two fig varieties, with different crop types, applying a multidisciplinary approach including anatomical, X-ray imaging, and genetic techniques, in order to better understand the mechanism behind the development towards breba or main (summer or winter) crop.

## Materials and Methods

### Plant Materials and Total RNA Extraction

Two fig varieties Dottato and Petrelli grown at the fig repository at the “P. Martucci” experimental station in Valenzano (Bari) of University of Bari “Aldo Moro” Department of Soil, Plant, and Food Science – Fruit Tree Unit, were used in this work. In particular, Dottato belongs to the Common type, which does not require pollination, and produces mainly main crop (rarely brebas) and the Petrelli belongs to the San Pedro type and produces mainly brebas and sometimes few main crop fruits. In 2016 and 2017, the two varieties were investigated for the caprification response whereas in the second year several approaches were undertaken to deeply study the buds. Moreover, total RNA from fruit buds of Petrelli and Dottato harvested in 2017 at two different time point, April and July (according to the development of the fruits, breba, and main crop respectively), was extracted according to the RNeasy Plant Mini Kit (QIAGEN^®^) instructions; for each stage and variety three different biological replicas were used, and for each of its three technical replicates. Considering that the standard deviation among the replicates was not significant, we used the mean for the subsequent analysis. RNA quality and quantity were assessed by Nanodrop 2000 (Thermo Scientific, USA) and checked on 1.5% agarose gel. RNA integrity was evaluated with Bioanalyzer 2100 and TapeStation 4200, only samples with a RIN higher than 8 were used for sequencing. After library construction, with fragment size of 275 bp and standard deviation of 166 bp, using a TruSeq Standard mRNA kit (Illumina USA), RNA sequencing was performed on a HiSeq 2000 Illumina system using a paired-end sequencing technique (2x100 bp).

### Genome Structural and Functional Annotation

The reference genome sequence of *F. carica* (Mori et al., 2017) was used as a basis for all the bioinformatics analyses, and it was downloaded from NCBI (accession number GCA_002002945.1). As a first step, a structural genome annotation was performed following the pipeline showed in **Figure S4**. Briefly, RNA-seq reads produced from this study were trimmed using BBDuk v35.85 (Bushnell et al., 2017) setting a minimum base quality of 20 and a minimum read length of 35 bp. Reads were then mapped against the reference genome with STAR v2.7.3a (Dobin et al., 2012) in double pass mode and the following parameters “–alignEndsType Local –alignEndsProtrude 20 ConcordantPair”. The resulting BAM files were used as input for Trinity v2.4.0 (Grabherr et al., 2011) transcriptome assembler in genome guided mode and using the following options “–jaccard_clip –min_kmer_cov 2 –SS_lib_type RF –genome_guided_min_reads_per_partition 10”. The raw assembly was processed with CD-HIT-EST v4.8.1 (Fu et al., 2012) to remove redundant sequences with the options “-r 0 -g 1” and then the assembly was evaluated with Transrate v1.0.3 (Smith-Unna et al., 2016) and BUSCOv3 (Simão et al., 2015).

The assembled transcript and the transcriptome sequences published in Solorzano Zambrano (Solorzano Zambrano et al., 2017) were used with the Maker2 v3.0.0 (Cantarel et al., 2008) pipeline to create a first annotation based only on expressed sequences. The obtained annotation was used to perform a training of an Augustus v3.3 (Stanke and Morgenstern, 2005) model which was then used with Maker2 to perform a new annotation iteration. The new obtained GTF was used to refine the training Augustus. This process was iterated 4 times before obtaining the final annotation. The newly obtained genome annotation was compared with the official one, to avoid loss of information the genes present in the official annotation were always maintained, in case of 1-to-1 overlap the official gene structure was maintained. In case of 1-to-many overlaps between our annotation and the NCBI annotation, a more in-depth analysis was performed: the corresponding proteins were BLASTed against TrEMBL and UniProt Plantae databases (April 2018) (minimum e-value 0.001) and the best proteins/genes were selected as the ones showing the highest alignment length. The predicted protein sequences were annotated with the AHRD v3.0 (https://github.com/groupschoof/AHRD) pipeline after performing a BLASTp against SwissProt and TrEMBL Plant sequences (April 2018).

### Quantification and Differential Gene Expression Analysis

Gene expression values were quantified for each sample with FeatureCounts v2.0 (Liao et al., 2013) together with the new genome annotation in order to calculate gene expression values as raw read counts. In addition, normalized TMM (Trimmed Mean of M-values) and FPKM (Fragments Per Kilobase per Million Mapped Fragment) values were calculated for all the genes.

The HTSFilter (Rau et al., 2013) package was chosen to remove the not expressed genes and the ones showing too much variability and implemented with a filtering procedure for replicated transcriptome sequencing data based on a Jaccard similarity index. The filter was applied to the different experimental conditions in order to identify and remove genes that appear to generate an uninformative signal. The TMM normalization was used for this step and the genes with TMM values lower than 7 were removed.

All the statistical analyses were performed with R with the packages HTSFilter (Rau et al., 2013) and edgeR (Robinson et al., 2009). Genes were considered statistically differentially expressed with an FDR value <=0.05.

### Identification of Differentially Expressed Genes

The sequences of genes involved in the complex system of flowering and fruit maturation reported in literature were downloaded from GenBank (http://www.ncbi.nlm.nih.gov/Genbank/) and *Morus notabilis* Genome Database (http://morus.swu.edu.cn/morusdb/). All the sequences obtained were used as queries to blast (http://blast.ncbi.nlm.nih.gov/Blast.cgi) against the yielded sequences from the *Ficus carica* RNA-seq (e-value threshold ≤E-10 and identity percentage higher than 80%). To determine the role in fruit ripening, differential expression analysis was carried out comparing the two varieties and the two different time points (fruits).

### Caprification Trial

Petrelli is a San Pedro-type variety defined as biferous since it needs caprification for the main crop, whereas Dottato, which is a Common-type fig defined as uniferous, does not need caprification. The trial was conducted in the fig repository of the Fruit Tree Unit - Department of Soil, Plant, and Food Science - University of Bari “Aldo Moro.” In this repository, trees are trained to vase and spaced at 6 × 6 m. The orchard is subjected to the cultural practices commonly adopted for fig trees in the area and weed management is done through mowing (no use of herbicides at all). The experimental plot consisted of 3 treatments with 3 biological replicates (trees) each: 1) open pollination of the fruits (T1); fruits covered with net without pollination, i.e., parthenocarpic (T2); fruit covered with net with hand pollination (T3). The shoots bearing fruits were covered with nets to prevent pollination by *Blastophaga psenes* (**Figure S5**). Hand-pollination was conducted every 4 to 5 days each year (2016–2017) by using a syringe with a thin needle. Pollen grains of the profichi were dispersed in solution containing 2% of sucrose and were injected in the fruits through the ostiole when ready to be pollinated (**Figure S6**). All the fruits were counted prior to bagging and successively at the end of the trial for calculating the percentage of fruit-set. At harvest, on the fruits that set, quantitative and qualitative measurements were made including size, weight, pH, total soluble solids (TSS, as °Brix), and titratable acidity (TA).

Analysis of variance (ANOVA) was performed with XLSTAT-Pro software (Addinsoft, Paris, France) at the 0.05 P level. The assumptions of variance were verified with the Levene test (homogeneity of variance) and the Lillefors and Shapiro-Wilk tests (normal distribution). The mean values obtained for the different factors were statistically separated by using the REGWQ test.

### Microscopic Bud Analysis

In 2017, 30 fruit buds and 30 mixed buds taken from the current year shoots were put in FAA (90% ethanol 50%, 5% acetic acid, and 5% formaldehyde) for 72 h. Buds were successively washed in distilled water and dehydrated in alcoholic solutions and finally included in metacrylate. The included buds were cross-sectioned by using a steel blade microtome (LKB Bromma 11800 Pyramitome). The sections of the buds were put on the slides and observed at the microscope (Nikon H550L).

In 2018, 10 fruit buds and 10 mixed buds were taken from the shoots every 3 weeks from summer (July) until end of winter (February) from each variety. External scales were carefully removed from each bud and sections (0.04–0.08 mm thick) were cut with a hand microtome and stained with 0.05% (w/v) toluidine blue. Images were acquired under a Nikon H550L light microscope (Nikon, Tokyo, Japan) equipped with a digital camera (Canon, Tokyo, Japan).

In order to better investigate the development of the different buds we set up a small experiment in a growth chamber at the end of the summer. Potted fig plants were placed in the growth chamber with controlled light (16/8) and temperature (26/22 °C). Bud burst of both brebas and apical buds was monitored for the successive months.

### X-Ray Analyses

High resolution micro X-ray computed tomography (µCT) analysis of fig buds was carried out at the MicroXRayLab of University of Bari “Aldo Moro.” Since sample dehydration during the scan strongly affects tomography data resulting in image distortions, fig buds were preliminary dehydrated with ethanol solutions with increasing concentration (up to 80% ethanol) as reported by Glauert (Glauert, 1974).

Each fig bud sample was glued at the tip of a wooden toothpick and analyzed with a SkyScan 1272 µCT (Bruker Gmbh, Germany), equipped with a W microfocus X-ray source operating at 40 kV and 250 mA. For all samples, a rotation step of 0.1 deg (0–180 deg) and an exposure of 1630 ms per radiography was set, for a total time of about 5-hours acquisition per sample. Image resolution (pixel size) varied between 1.6 and 2.1 µm, depending on sample shape and dimension. Flat field correction, frame averaging (3) and random movement (10) were also applied to improve the accuracy of the results.

After acquisition, image reconstruction was carried out using the software NRecon (version 1.6.10.4, InstaRecon^®^), while for the 3D rendering the software DataViewer (version 1.5.2.4, Bruker microCT^®^) and CTvox (version 3.1.1 r1191, Bruker microCT^®^) were jointly used.

## Results

### Genome Structural and Functional Annotation

The genome of *F. carica* was published in 2017 (Mori et al., 2017), which reported 36,138 gene models, 69% of which were functionally annotated. Own transcriptomics data and publicly available data were used to update the annotation with variety specific expressed genes that might not have been represented in the official one. In order to do so, the approach described in **Figure S4** was adopted. Briefly, a reference guided transcriptome assembly produced 50,866 transcripts with a mean length of 875.16 bp. The dataset used for the assembly included 167 million of paired-end 100 bp reads from six samples. Analysis performed with the Viridiplantae dataset of BUSCO conserved genes found that the assembled transcriptome included 76.6% of complete genes (i.e., single copy and full length) (**Figure S7**). The assembled transcripts, together with the transcripts from the official annotation and a *de novo* assembly produced by Solorzano Zambrano (Solorzano Zambrano et al., 2017; Usai et al., 2020) were used with the Maker2 pipeline to produce a pre-annotation that was then used to create an Augustus gene model. The model was refined 4 times before the genome annotation was obtained, which contained 35,567 genes. About 34,600 gene models were in common between the official annotation and our version. In addition, 938 new genes were annotated and 1,509 genes from the Mori et al. (2017) annotation were removed since they were gene fusions or gene fragments (see *Methods*). The BUSCO analysis (**Figure S7**) revealed that 79% of genes were complete in the new annotation, 68.8% were also in single copy whereas 10.3% were duplicated. On the other hand, the official annotation contained 67.3% of single copy genes and 10.2% of duplicated genes. In addition, the new annotation contained 6.18% of fragmented genes against the 6.45% of the reference. Finally, the percentage of missing genes was 14.5% in the new annotation and 15.9% in the reference. A description was assigned to 25,910 genes (72.84% of the total) whereas a Gene Ontology annotation was assigned to 24,987 genes (70.25% of the total).

### Differential Gene Expression Analysis

A total of 35,567 (34,629 known and 938 novel genes) protein-coding genes were predicted in the genome assembly. Among them, 21,762 and 21,441 were expressed in Dottato fruits in brebas and main crop, respectively, while fruits of Petrelli had 21,801 and 21,852 genes expressed in brebas and main crop, respectively (**Figure S8**). Transcripts with expression values of zero in all the samples were not considered into the analysis. Among the total of expressed loci, in breba 1,072 and 1,111 loci were specific for Dottato and Petrelli, respectively, whereas 20,690 genes were expressed in both varieties. In the main crop, instead, 992 and 1,403 genes were detected in fruits of Dottato and Petrelli, respectively. Both varieties shared 20,449 expressed loci (**Figure S9**).

Within the 1,615 and 1,904 loci expressed in Dottato and Petrelli specifically in breba or main crop, respectively, only 256 genes appeared to be transcripts in both varieties. The other loci were clearly specific for each variety and in particular 1,359 loci for Dottato and 1,648 genes for Petrelli (**Figure S9**).

Two databases (KEGG and GO) were used to annotate all unigenes with comprehensive gene function information. A total of 7,056 genes were successfully annotated using KEGG database, representing the 19.8% of the total. The KEGG database identified the putative biological pathways of genes (**Table 1**) with a total 130 genes expressed in Dottato (66 loci in breba and 64 loci in main crop) and 203 transcript specific in Petrelli (95 loci in breba and 108 in main crop). With respect to the KEGG pathway distributions, SAUR (Small Auxin Up RNAs) family proteins, followed by transcription factor MYB and interleukin-1 receptor-associated kinase 4 were the most common. The signaling molecules and the sugar metabolizing enzymes had a low gene count (two or three genes per pathway).

**Table 1:** Comparative analysis of differentially expressed genes between the two stages of bud development in Dottato and Petrelli, the number of genes annotated in KEGG database and its specific description.

Variety	No. of KEGG annotated genes	Most represented KEGG pathways	
		No. of loci	Description
Dottato (Breba)	66	2	Ferric-chelate reductase
		2	Glycerol-3-phosphate acyltransferase
		2	Solute carrier family 15 (peptide/histidine transporter), member 3/4
		3	Beta-glucosidase
		3	Laccase
		5	SAUR family protein
Petrelli (Breba)	95	2	Galacturan 1,4-alpha-galacturonidase
		2	Interleukin-1 receptor-associated kinase 4
		2	Disease resistance protein RPM1
		2	HSP20 family protein
		2	Omega-hydroxypalmitate O-feruloyl transferase
		2	Cytochrome P450 family 714 subfamily A polypeptide 1
		3	Peroxidase
		3	Pectinesterase
		4	Transcription factor MYB, plant
		10	SAUR family protein
Dottato (Main crop)	64	2	Galacturan 1,4-alpha-galacturonidase
		4	SAUR family protein
		5	Transcription factor MYB, plant
Petrelli (Main crop)	108	2	Endoglucanase
		2	Polygalacturonase
		2	Laccase
		2	Indole-3-pyruvate monooxygenase
		2	Calcium-binding protein CML
		2	ABA-responsive element binding factor
		2	SAUR family protein
		2	Solute carrier family 15 (peptide/histidine transporter), member 3/4
		2	Cytokinin riboside 5′-monophosphate phosphoribohydrolase
		3	Nuclear transcription Y subunit beta
		3	MADS-box transcription enhancer factor 2A
		4	Peroxidase
		4	Transcription factor MYB, plant
		5	Interleukin-1 receptor-associated kinase 4

The GO database assigned a predicted function to 24,987 genes (70.2%), including biological process, cellular component and molecular function. Among the annotated genes, 9,611 (27%) were found differentially expressed, and specifically 4,664 (13.1%) were up-regulated, while 4,947 (13.9%) were down-regulated in both Dottato and Petrelli. As a following step, a deferential expression analysis using separately the two groups of the up- and down-regulated genes was performed comparing Dottato vs. Petrelli. The evaluation underlined how 15 genes were up-regulated of which 10 genes were responsible of molecular function, 2 for cellular component, and 3 coding for biological processes (**Figure S10A**), with the signal transduction pathway (3–5 genes) the most represented. Analyzing the down-regulated genes, 20 were differentially expressed in the two varieties, and in details 12 corresponded to molecular function, 1 for cellular component, and 7 to different biological processes. Even in the down-regulated transcripts, the signal transduction pathway genes (2–3 genes) were the most common (**Figure S10B**).

### Transcriptome Analysis of Flowering Genes During Bud Differentiation

To find the main genes potentially involved in the flowering process and in the bud differentiation of main crop or breba, we used our transcriptome as a query to blast and select candidate genes.

Gene families including sequences of *MADS-box* (comprising *Agamous*, *AG*, and *Defieciens*, *DEF*, genes), flowering time control protein (*FCA*, *FPA*, and *FY*) and *Apetala* (*AP*), which are responsible of the flowering, and the hormones, such as auxin (*AUX*), gibberellin (*GA*), ethylene (*ETH*) and indole-3-acetic acid (*IAA*), which regulate the plant growth, were identified in NCBI and *Morus* (phylogenetically closest genus belonging to the *Moraceae* family) database and the expression levels of the correspondent transcripts were compared in both Dottato and Petrelli fruit types.

The blast analysis against the *Ficus carica* annotated genes and the homologue sequence from *Morus* database, allowed the identification of 151 loci associated with genes for either flowering or hormones for plant growth. Among those, 5 loci were obtained from the *Ficus* genome, instead 146 genes were detected through *Morus* database. In particular, the most represented classes were auxin and ethylene gene families with 54 and 45 transcripts, respectively, followed by gibberellin (16 genes) (**Table S1**).

To explore the flowering genes, the expression in the two crops (breba and main crop) of all genes obtained from this analysis was examined and compared between the two varieties. The expression patterns of some of the genes following the auxin, ethylene, and gibberellin groups significantly differed between Dottato and Petrelli. Looking at the genes primary implicated in the flowering process eighteen genes were identified. Among them, the results showed high variability not only between but also in the same varieties for breba and main crop. In particular flowering locus T (*FcFT*), flowering time control protein (*FCA*), and flowering time control protein (*FPA*) had the highest expression level in the main crop of Petrelli; floral homeotic protein AGAMOUS (*AG*), floral homeotic protein APETALA (*AP2*) and flowering time control protein (*FY*) were more expressed in breba of Dottato; 1-aminocyclopropane-1-carboxylate oxidase-like 3 (*ACO3*) and floral homeotic protein APETALA (*AP1*) had high values of expression in main crop of Dottato and breba of Petrelli, respectively (**Table 2**).

**Table 2:** Expression patterns of the genes primary implicated in the flowering process significantly different between Dottato and Petrelli.

Gene	Enzyme	Source		Ficus Locus	Expression level				Differential expression
		Specie	Accession number		DB	PB	DM	PM	
*Ficus carica*									
*FcFT*	flowering locus T		AB594722.1	s01389g28557	12.1	1.5	2.1	30.3	–
*ACO2*	1-aminocyclopropane-1-carboxylate oxidase 2		KP892660.1	s00021g02797	0.1	0.1	0.0	0.3	–
*ACS4*	1-aminocyclopropane-1-carboxylate synthase 4		KP892659.1	s00001g00020	3.0	2.3	3.1	8.1	–
*ACO3*	1-aminocyclopropane-1-carboxylate oxidase-like 3		KP892661.1	s00803g24021	11.3	3.4	34.6	18.8	–
*ACS1L*	1-aminocyclopropane-1-carboxylate synthase-like 1L		KP892658.1	s00311g15561	0.4	0.0	0.4	3.7	–
*Morus notabilis*									
*AG*	Floral homeotic protein AGAMOUS		EXC21999.1	s00824g24272	113.0	78.6	22.9	31.2	Up
			EXC21999.1	s00026g03239	145.3	116.3	43.6	26.9	Up
*AP1*	Floral homeotic protein APETALA 1		EXB44879.1	s00016g02309	661.8	823.9	353.3	472.1	–
*AP2*	Floral homeotic protein APETALA 2		EXB30380.1	s00088g07632	0.2	0.3	0.2	0.4	–
			EXB30380.1	s00991g25958	0.4	0.6	0.2	0.0	–
			EXC24730.1	s00433g18352	118.1	94.2	36.1	46.5	Up
			EXB84815.1	s00019g02645	123.5	121.7	54.6	33.5	Up
*DEF*	Floral homeotic protein DEFICIENS		EXC35487.1	s00151g10560	0.1	0.1	0.0	0.0	Up
			EXB48382.1	s00042g04637	6.0	6.9	1.8	1.1	Up
*FCA*	Flowering time control protein FCA		EXC27503.1	augustus_masked-BDEM01000163.1-processed-gene-1.14	43.3	38.0	41.6	58.7	–
*FPA*	Flowering time control protein FPA		EXB23115.1	s00987g25910	12.3	9.4	16.6	20.7	Down
			EXC35026.1	s00385g17337	12.5	11.9	14.6	15.4	–
*FY*	Flowering time control protein FY		EXB62656.1	augustus_masked-BDEM01001238.1-processed-gene-0.1	26.8	19.9	23.2	24.5	–

DB, Dottato Breba; DM, Dottato Main crop; PB, Petrelli Breba; PM, Petrelli Main crop.

No detectable variation in the expression pattern was observed for the following genes between breba and main crop and between the two varieties: 1-aminocyclopropane-1-carboxylate oxidase 2 (*ACO2*), 1-aminocyclopropane-1-carboxylate synthase 4 (*ACS4*), 1-aminocyclopropane-1-carboxylate synthase-like 1L (*ACS1L*), two of the *AP2* and Floral homeotic protein DEFICIENS (*DEF*) (**Table 2**).

### Caprification

In the year 2016, fruit-set was variable as a function of the flights of *Blastophaga psenes* for both the climatic conditions and the number of individuals in the fruits. In the case of open pollination, in Petrelli fruit-set was 66.7% and in Dottato 90.9%. Similar values were obtained by hand-pollination under the net, with 57.1 and 95.0% for Petrelli and Dottato, respectively (**Table 3**). When pollination was prevented to obtain parthenocarpic fruits, fruit-set in Petrelli was only 12.0% but fruit set in Dottato was not affected (93.4%).

**Table 3:** Effects of open, hand and no pollination (parthenocarpic) on fruit-set (%) of Petrelli and Dottato for 2016 and 2017.

Treatment	2016		2017	
	Petrelli	Dottato	Petrelli	Dottato
**Open pollination**	66.7a	90.9	81.5a	100
**Parthenocarpic**	12.0c	93.4	28.6b	100
**Hand pollination**	57.1b	95.0	86.4a	100

Small letters indicate significant differences (P < 0.05) between treatments within each year and variety according to REGQW test.

In 2017 data were different in the number but not in the trend, maybe because more caprifigs were used for releasing the pollen grains. In particular, fruit-set was always 100% in Dottato, for all the treatments. In Petrelli, fruit-set was higher than the previous year, with 81.5 and 86.4% for open and hand pollination, respectively, but was only 28.6% in non-pollinated (parthenocarpic) fruits (**Table 3**).

Quantitative and qualitative traits showed differences between the pollinated and non-pollinated fruits (**Table 4**). Hand-pollinated Petrelli and Dottato fruits were heavier than open pollinated or parthenocarpic fruits (**Table 4**). Differences arose also for size, with hand pollinated Dottato fruits higher than fruits from the other treatments; hand pollinated Petrelli fruits resulted also larger than fruits of the other two treatments (**Table 4**). Hand pollinated fruits of Dottato ripened earlier than non-pollinated fruits (5–6 days) with higher total soluble solids (TSS) than fruits from other treatments; but TA and pH of hand-pollinated fruits were lower and higher, respectively, than parthenocarpic fruits. The pulp of hand pollinated Dottato fruits was less juicy and with an intense red color and with a large number of drupelets, whereas in the open pollinated and parthenocarpic fruits the color of the pulp was lighter (**Figure S11**). In the case of Petrelli fruits, the lower titratable acidity (TA) was noticed for hand pollinated fruits which also had a higher pH with respect to open pollinated and parthenocarpic fruits (**Table 4**). The taste of the three types of fruits was almost identical.

**Table 4:** Effects of open, hand, and no pollination (parthenocarpic) on fruit quality of fruits of Petrelli and Dottato.

Treatment	Weight (g)		Height (mm)		Width (mm)		TSS (°Brix)		TA (g/kg)		pH	
	Petrelli	Dottato	Petrelli	Dottato	Petrelli	Dottato	Petrelli	Dottato	Petrelli	Dottato	Petrelli	Dottato
**Open Pollination**	57.7b	58.7b	45.8	52.9b	47.3b	49.5	19.8	23.3b	1.26a	1.22c	5.47b	5.80a
**Parthenocarpic**	60.4b	55.0b	47.6	50.8b	49.7b	48.4	19.6	22.3c	1.22a	2.14a	5.47b	5.00c
**Hand pollination**	80.5a	64.4a	48.8	61.7a	55.1a	50.1	19.1	24.7a	1.10b	1.70b	5.57a	5.28b

Small letters indicate significant differences (P <0.05) between treatments within each year and variety according to REGQW test.

### Microscopic Buds Analysis

Buds taken from the shoots showed some differences mainly related to the stage of the development, less developed in the distal nodes with respect to the more developed buds at the basal nodes of the current year shoot. In Petrelli, the fruit buds in the distal nodes showed small, elongated and curved ostiolar scales (**Figure 1A**), whereas in the basal fruit buds the ostiolar scales were much larger with cells more developed (presence of florets) in the cavity of the syconium (**Figure 1B**). In Dottato, differences in the development of the fruit buds were also evident; in the smaller fruit buds at the apical position the ostiolar scales were still underdeveloped, and the florets were almost invisible (**Figure 1C**), whereas at the basal nodes buds had ostiolar scales completely developed, and the structures of the florets resulted also visible (**Figure 1D**).

The evolution of brebas buds from the dormant season to summer is clearly visible for both Dottato (**Figure S12**) and Petrelli (**Figure S13**). The greatest development of the brebas of Dottato was between March-April. Similar growth was observed for Petrelli, with another smaller size increase in May-June (**Figures S12** and **S13**).

**Figure 2:**
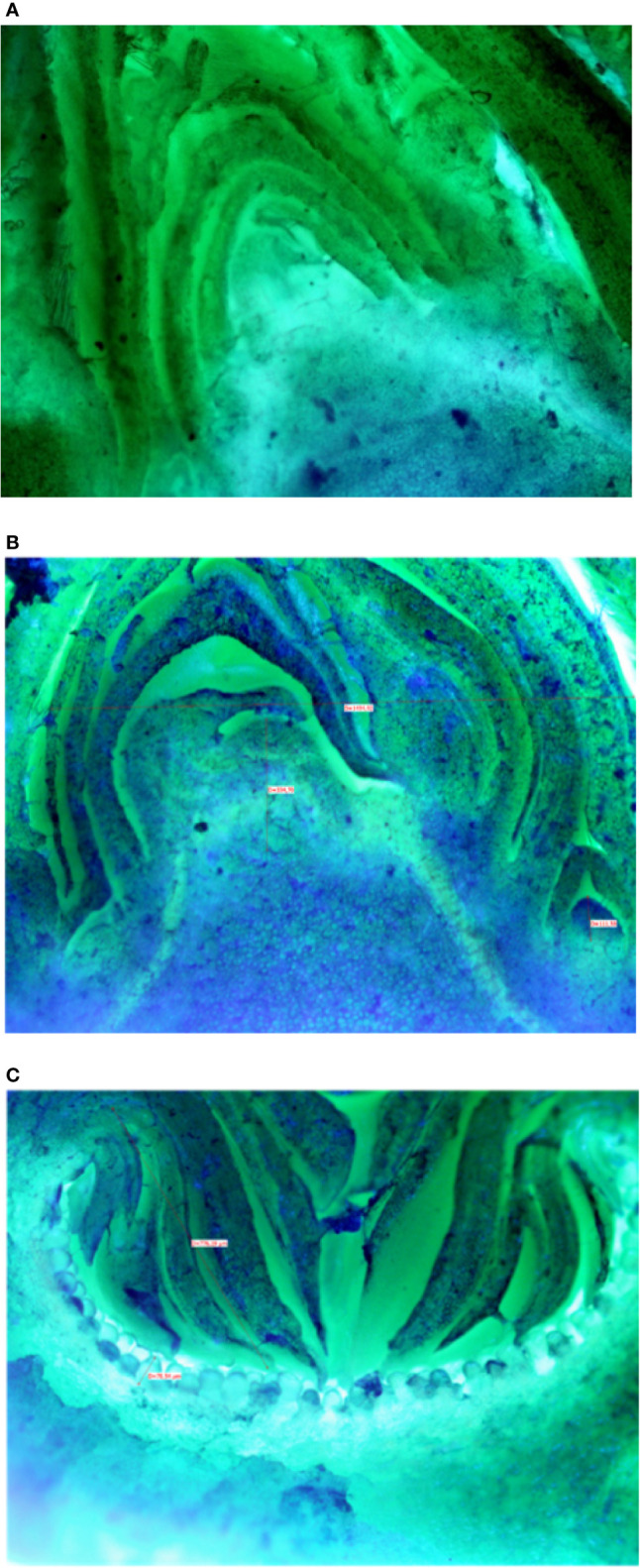
Sections of: the apical bud (mixed) with no signs of inflorescence differentiation in December **(A)**; the apical bud (mixed) with small-undifferentiated inflorescence at the basal node **(B)** in February; a fruit bud (breba) already differentiated **(C)** with florets in the receptacle in summer (August-September).

Microscopic analyses of buds from July until February showed that differentiation of flat receptacles had not occurred at apical/lateral mixed buds until December (**Figure 2A**). Apical/lateral buds showed small undifferentiated inflorescences (main crop) at the basal-nodes of the meristem but not at the apical nodes by the end of January (**Figure 2B**). Conversely, the brebas had already formed florets (not completely differentiated) in July, which were characterized by masses of cylindrical pistillate primordia, and remained at this stage from summer until the end of winter before bud burst (**Figure 2C**). To our knowledge, this was the first time such a long survey of bud development in *Ficus carica* was conducted either on apical/lateral buds or fruit buds. The sections of fruit and mixed buds of Dottato and Petrelli are shown from July to February in **Figures S14** and **S15**, respectively.

### X-Ray Analyses

**Figure 3:**
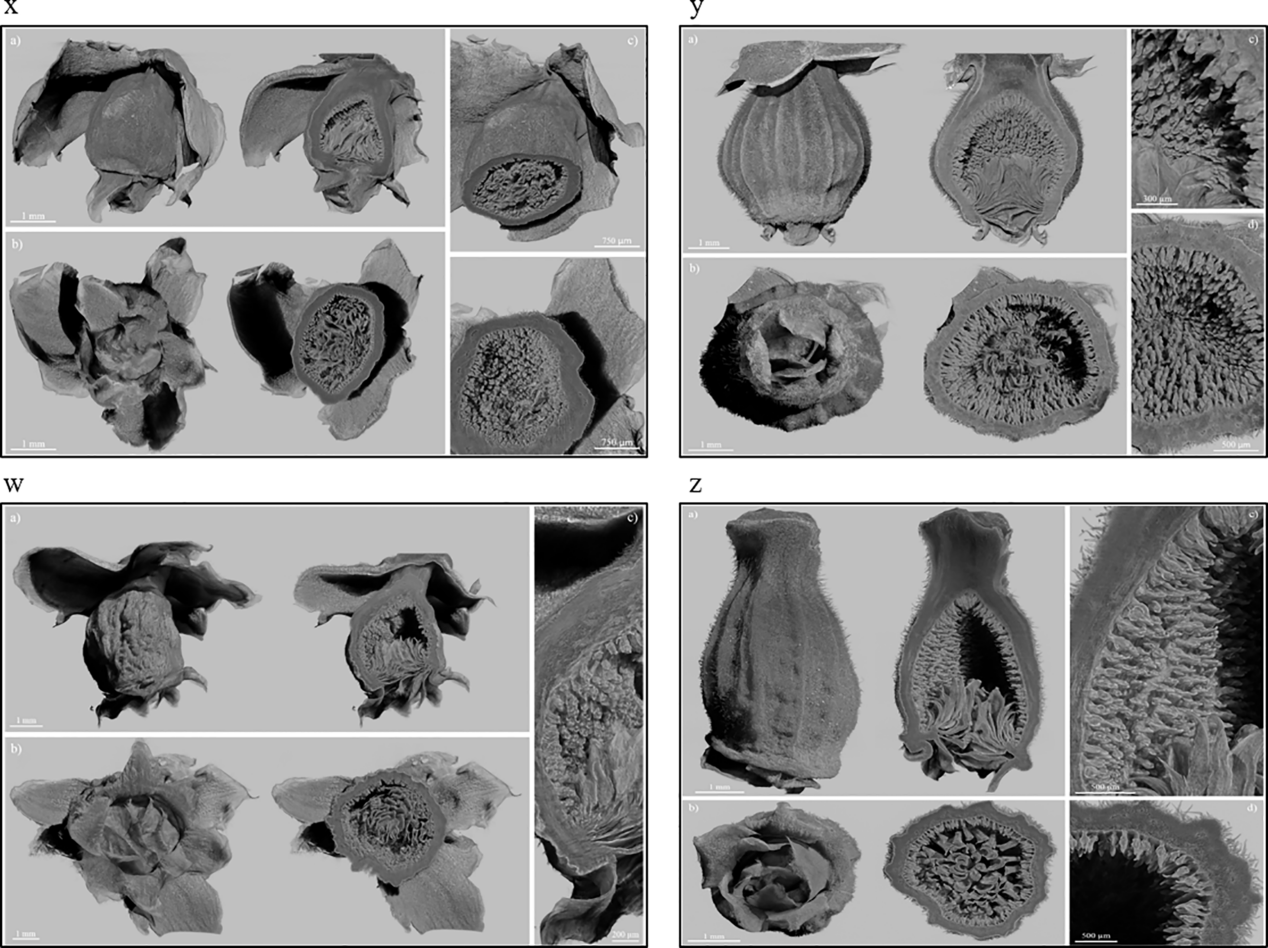
Fruits of Dottato, breba **(x)** and main crop **(y)**, and Petrelli, breba **(w)** and main crop **(z)**, as obtained by X-ray analysis. For each panel, subfigures a) and b) show the frontal and bottom views, respectively, with related internal cross sections; subfigures c) and d) show internal details.

The X-ray images of Dottato showed a great structural similarity between the breba (**Figure 3**x) and the main crop (**Figure 3**y). The two fruits were taken at the same size and, as shown by the microanalyses, the two structures are very similar. The ostiolar scales are well developed (**Figure 3**x, y a) and completed their development before the final growth of both the receptacle and the florets. The fruit bud of the breba is covered (**Figure 3**x b) with several bud scales, since it is an overwintering bud, whereas the fruit bud of the main crop (**Figure 3**y b) develops on the current season’s shoot. In the case of Petrelli, **Figures 3**w–z show the two structures which differentiate either for the bud scales, more abundant in the case of the bud of the breba (**Figure 3**w) or for the more elongated shape of the main crop (**Figure 3**z). Ostiolar scales and the florets in the receptacle are almost identical in the two crops, which make them almost undistinguishable from an anatomical point of view in the first developmental stages.

## Discussion

### Molecular Analyses

In the current study, genes involved in the natural process of flowering and flower differentiation in breba or main crop of fig were investigated. The expression of fig flowering-related and hormone genes was examined to understand their functions in fig fruits and further the regulation mechanisms in this unique species. In particular, we were able to identify twelve genes expressed in the two crops (breba and main crop) and between the two varieties analysed (Dottato and Petrelli) involved in the formation of organs of the fig inflorescence (sepals, petals, stamens, ovary). Among those genes, some of them showed a differential expression pattern underlining the putative role of each of them in flowering process. In particular, the *flowering locus T* gene, which showed high expression level in the main crop of Petrelli, has been identified in other species (e.g., *Arabidopsis thaliana* and cucurbits) as a key gene in the regulation of flowering, in relation to day length changes, (Turck et al., 2008) and floral induction (Lin et al., 2007). Several reports have identified other genes related to flowering, including the *APETALA1* gene (which appeared to be differentially expressed in our analysis) in activating the production of reproductive organs (Abe et al., 2005; Teper-Bamnolker and Samach, 2005; Wigge et al., 2005; Amasino and Michaels, 2010). Similar mechanism has been proposed for the *APETALA2* gene (*AP2*), which in *Arabidopsis* promotes early floral meristem identity (Jofuku et al., 1994) and is required for the transition of an inflorescence meristem into a floral meristem (Drews et al., 1991). In addition, AP2 takes part in the specification of floral identity in *Arabidopsis* plant (Drews et al., 1991; Krogan et al., 2012).

A large number of genes following the flowering time control family (including *FCA*, *FPA*, and *FY* identified in our work) have been identified in grapevine (Kamal et al., 2019), as the main genes involved in the high complex flower development and long duration of bud initiation, and in *Arabidopsis thaliana*, in which promote the transition of the vegetative meristem to reproductive development.

In our analysis, we detected, also, the AGAMOUS gene, which appeared to be highly expressed in Dottato compared to the Petrelli, especially in the brebas (parthenocarpic fruits). In *Arabidopsis* this is a transcription factor involved in the control of the organ identity at the early stages of flowers development and interacts genetically with the other homeotic genes such as APETALA2 (Yanofsky et al., 1990; Bowman et al., 1991a; Bowman et al., 1991b; Drews et al., 1991). APETALA2 resulted also upregulated in Dottato with respect to Petrelli, and in general, AGAMOUS, APETALA1, and APETALA2 resulted more expressed in brebas than in the main crop.

Interestingly, we detected a differential expression level of some *1-aminocyclopropane-1-carboxylate oxidase* (*ACO*) genes, with the *ACO3* the most interesting, showing high expression amount in the main crop of Dottato. Apparently the ACO family is not directly related with the flowering process, but catalyzes the final step of ethylene biosynthesis (Houben and Van De Poel, 2019), a pathway usually connected to climacteric fruit ripening and senescence and one of the primary targets of biotechnology for increasing the shelf life of vegetables and fruits. Nevertheless, some studies have demonstrated that *ACO* gene regulation can affect flower formation and development (Houben and Van De Poel, 2019). In detail, the silencing of *ACO* genes can delay flower senescence and abscission in petunia, carnation, and torenia (Savin et al., 1995; Aida et al., 1998; Huang et al., 2007; Tan et al., 2014). Additionally, a mutation of the *ACO2* gene in cucumber affected the sex determination, producing only male flowers (Chen et al., 2016).

Ikegami (Ikegami et al., 2013) reported that FcFT1 mRNA levels were higher in basal nodes (main crop) with respect to distal and younger nodes (breba) thus suggesting a partial correlation between inflorescence differentiation and FcFT1 expression. We observed a higher expression of FT in the main crop of Petrelli with respect to the other fruits. Receptacles with florets differentiated at the same time as FcFT1 expression levels begin to increase, supporting a relationship between FcFT1 continuous expression and flowering as well as fruiting (Ikegami et al., 2013). FcFT1 expression activated by light is indispensable to fruit bearing, including inflorescence differentiation, and the particular two crops of fig (main and breba) in two distinct seasons are probably due to the long-term stable expression of FcFT1 (Ikegami et al., 2013).

We found a higher expression of several genes related to auxin (auxin efflux carrier, auxin response factor, auxin binding protein, auxin responsive protein) and to GA synthesis (GA20ox) in brebas with respect to main crop. Auxin and gibberellin content was higher in brebas than in the main crop of San Pedro cv. King (Lodhi et al., 1969; Chai et al., 2017) to support the growth of parthenocarpic fruits and expression of GA and auxin-biosynthesis gene is repressed in main crop of San Pedro (seeded fruit) with respect to breba, whereas ABA and ethylene-biosynthesis genes are enhanced (Chai et al., 2017). In particular, genes related to GA synthesis (GA20ox) were upregulated in brebas of San Pedro fig at the stages of anthesis and post-anthesis whereas ABA genes were highly expressed in the main crop fruits (Chai et al., 2017).

### Caprification

When Petrelli fruits were caprificated (open or hand pollination) fruit-set was always higher than in non caprificated fruits. In contrast, Dottato had high fruit-set regardless of how it was pollinated. Better results (higher fruit-set) were achieved in the second season when pollen was collected from more caprifigs. Important characteristics of the caprifigs (profichi crop) such as fruit size, fruit number per shoot, amount of pollen production and pollen viability can affect the fruit-set and successive quality of fruits; thus, caprification with different types of caprifigs will give different results in terms of fruit-set, yield, and quality as indicated by the data of the second season.

The lower fruit-set of 2016 for the open pollination treatment may be due to the lack of caprification or to the deficiency of caprification caused by weather conditions such as temperature and wind, which can reduce pollinator activity (Oukabli et al., 2003). The differences in hand pollinated fruits collected in 2016 and 2017 are the consequence of the pollen sources used in the two years. In Tunisia, different caprifigs affected both the fruit-set and qualitative parameters such as fruit size, skin thickness, flesh thickness, seed number, TA, and TSS (Gaaliche et al., 2011c). Similarly, in Iran pollen of different caprifigs had a significant effect on fruit length, TSS, ostiole diameter, and percentage of seed germination (Rahemi and Jafari, 2008). Another study conducted in Iran on the cvs Payves and Sabz with three different caprifigs (Avgeizi, Sarbasteh, and Kouhi) demonstrated that the pollen source had significant effect on fruit length, skin color, total soluble solids, total phenolics, total flavonoids, and total anthocyanins (Pourghayoumi et al., 2012). The caprification intensity and more than one type of caprifig in the orchard can either extend the caprification period or affect quality and yield of the main crop (Mars et al., 2017). The differences between hand-pollinated and open-pollinated fruits could be ascribed to both the amount and intensity of pollination. In the case of open-pollinated fruits, the profichi fruits of the three types of caprifigs in the fig orchard were left hanging on the caprifigs, whereas when hand-pollination was accomplished, pollen grains from the profichi of the three caprifigs, were collected and added in the sucrose mixture injected with the syringe directly in the fruit cavity. The mixture of pollen grains used for hand-pollination may have enhanced some characteristics of the fig fruits with respect of the pollen grains carried by the wasps from the profichi fruits in the orchard.

In Turkey, other authors reported that pollinated fruits were distinguished by superior quality (higher TSS, firmness, and more intense external and internal color) and could be better stored than parthenocarpic ones (Aksoy et al., 2003). In our case, better results were obtained with hand pollination of Dottato fruits which ripened earlier and had a red colored pulp. In general, pollination affected the quality parameters of the fruits in particular fruit size and weight (Gaaliche et al., 2011a) but, being pollinated or not, figs develop almost the same concentrations of aroma when ripe and non-pollinated fruits tend to develop more ketones and alcohol compounds (Trad et al., 2012). However, some compounds responsible for pleasant aromas and flavors degenerate when figs reach maturity without being pollinated; that was noticed in the case of butyl and hexyl acetate and beta-ionone in Bouhouli fruits (Trad et al., 2012).

In the case of Brown Turkey figs, ripening pollinated fruit differed from parthenocarpic ones in their shape, which was round for the former and pear-shaped for the latter (Rosianski et al., 2016). Similarly to our results for Dottato (common type), pollinated Brown Turkey figs were heavier than parthenocarpic figs and also larger (Rosianski et al., 2016), whereas Dottato pollinated fruits resulted higher and heavier than parthenocarpic ones in our study. The different color of the pulp between pollinated and parthenocarpic figs was also clearly observed for the inner inflorescence of Brown Turkey throughout fruit development. In particular, in parthenocapric fruits anthocyanin level reached the values of pollinated fruits in much later stages (Rosianski et al., 2016). In general, pollinated ripe fig fruits are much better than parthenocarpic fruits in growth, width, weight, firmness and taste qualities (Rosianski et al., 2016).

### Buds Analyses

The analyses of the buds at the beginning of the season, when buds were small and in the growing stage, showed significant differences in the development of the basal buds with respect to the distal buds. The basal buds are devoted to develop the main crop, the middle buds main (late) crop and the distal nodes bear brebas. The different development of the buds can be explained by their different evolution, development in the current season for the basal buds/main crop and development in the successive season for the distal buds/brebas. We observed that from July onward, the distal buds had already developed florets in the receptacle and remained in a dormant stage during autumn-winter time. Apical/lateral mixed buds showed an undifferentiated apex, thus suggesting the differentiation of the inflorescences (breba and main) occurs in the successive growing season. However, at the lower nodes of the apical/lateral buds the presence of undifferentiated primordia was visible at the end of winter (January-February), but no signs of primordia were visible in the middle-distal nodes. It is clear that the inflorescence formation and differentiation process occurs sequentially, from the basal nodes (main crop) toward the middle (main crop-brebas) and distal ones (brebas). The formation of the primordia (main crop) in the basal nodes of the meristem started at the end of winter when temperature arose and successively proceeded during the season to the distal nodes.

With regards of the potted fig plants in the growth chamber, the controlled climatic conditions did not stimulate the development of the distal fruit buds (brebas) which stayed dormant for the successive months. Conversely, light and temperature induced the bud burst of either vegetative or mixed buds with the latter developing main crops on the growing shoots (unpublished data). Environmental factors (light, temperature) were not the limiting factors for fruit buds to burst but probably some endogenous factors (hormones, amino acids) or a limited cold requirement could play a major role for the activation of genes involved in fruit buds burst. The fruit buds of the distal nodes are covered with scales to pass dormancy and protect from winter injury, so they are prone to overwinter instead of developing in the current season although light and temperature were not a limiting factor in the growth chamber. Chill requirements for these fruit buds, although low, were not probably satisfied, and they did not burst. Vegetative and mixed buds reacted to the climatic conditions and burst with the formation of new shoots and developing main crops. In summer in the field, all the buds on the current year shoot are differentiated but some of them enlarge and grow as main crop and others do not and will develop (or drop or become latent) in the successive year as brebas.

The most relevant fruit tree species such as sweet cherry, apple, peach but also grape have flower (mixed) buds which partially differentiate during the summer of the previous year (such as the fruit buds of brebas) and the process is completed at the beginning of the successive season (spring) for a two-year process (Koutinas et al., 2010). In the case of figs, the distal fruits buds (brebas) differentiate in two seasons (spring-summer of year 1 and spring of year 2, such as the inflorescence of grape) whereas inflorescence of the main crop differentiates in the same year of formation since at the end of winter only rudimentary primordia are visible in the basal nodes of the apical/lateral mixed buds. In this study, we showed the differentiation started at the basal nodes at the beginning of the growing season and proceeded in the successive nodes during the season. The singularity of the fig is that only some differentiated inflorescences develop in the season (main crop) whereas others, start to differentiate (florets present), but do not continue to grow and develop during the current season. Molecular analyses also indicated a higher expression level of APETALA1 and APETALA2, AGAMOUS genes involved in flower organ formation, in brebas of both Dottato and Petrelli with respect to the main crop. The higher expression of AGAMOUS (required for stamen development) in breba may suggest a possible presence of staminate flowers at the ostiole of brebas in the origin of fig during its evolution. Higher expression of auxin and gibberellin genes in brebas may confirm this hypothesis, since brebas are parthenocarpic (persistent) fruits with no seeds and hormones are necessary for development and ripening of fruits. When the fig was monoecious, the growth of brebas was probably necessary to allow pollen dispersal of male flowers by the wasps, as in the profichi of the caprifig. AGL11 highly expressed in brebas and upregulated in Dottato is a possible candidate gene for parthenocarpy of fig fruits (both breba and main crop) as for seedlessness in grape (Mejía et al., 2011). Moreover, the ethylene-synthesis gene ACO3 was more expressed in main crop fruits with respect to breba fruits for the ripening of climateric fruits.

A study conducted on five varieties grown in Tunisia showed a growth model with two main vegetative growth flushes, with the first much longer than the second (Gaaliche et al., 2011b). The second flush, generally occurring at the end of summer, is strongly influenced by the climatic conditions, but it never lasts after the end of the summer (Gaaliche et al., 2011b). The buds of brebas are located in the distal portion of the shoot that develop late in the growing season, with respect to the portion where main crops are borne, and these buds may be more susceptible to erratic climatic conditions at the end of summer. In a three-year study, the main crop amount exerted a strong influence on the fruiting intensity of the successive year; in particular, a heavy main crop load decreased the number of brebas in the following year, while a heavy breba crop load reduced the main crop load of the current season (Gaaliche et al., 2011b). In the case of heavy main crop, the number of fruit buds (brebas) which remain dormant is clearly lower since almost all buds on the current shoot developed during the season. Conversely, the higher number of fruit buds (brebas) retained on 1-year shoots and developing in the successive season will compete with both the current year shoot growth, and the main crop fruits thus reducing the yield of the main crop and the shoot length. The behavior is an alternate bearing as in many tree fruit species to balance between vegetative and reproductive activity.

In this study, in apical (mixed) buds before bud burst (from July of year 1 until March of year 2) we did not observe inflorescence primordia (pistillate florets) but only a rudimentary undifferentiated structure until the end of winter. The mixed bud will differentiate first inflorescences (lower nodes, main crop) in spring of year 2. The flower primordia will completely differentiate in spring-summer both for the main crop and the brebas on all the nodes of the new shoot, since florets are already present in the fruit buds (brebas) in July in the distal nodes of the shoot (**Figures S14** and **S15**).

Moreover, X-ray images showed brebas and main crop were very morphologically similar at the first stages of development with the difference only in the number and thickness of bud scales. The two types of crops are almost undistinguishable at the very beginning of the structure enlargement. It seems that the buds of the brebas are prone to pass a dormant period (number and thickness of scales), whereas the fruits of main crop have lighter scales because have to develop in the current season but may ripe very late in the season (autumn-winter) like the mamme fruits of the caprifig.

Phylogenetic studies have shown that the common fig has a monoecious ancestor (Machado et al., 2001) and successively evolved in a gynodioecious species. The presence of these dormant fruit buds evolving in brebas at the beginning of the season may be a relic of the ancient monoecy, with the wasps entering the different inflorescences (main summer crop, main late crop, breba) during the year to lay the eggs, as they nowadays do in the caprifigs. In fact, the primitive condition in the mutualism fig-wasp, dating back ca. 90 million years ago, was the monoecius breeding system in the fig and the passive pollination in the wasp which was useful for either the fig (seeds) and the wasp (offsprings) (Machado et al., 2001). The particular bud evolution of the fig for main crop (in the current season) and breba (in the successive season) may be because the mutualistic evolution with the wasp in order to host the different generations to both pollinate and ovoposit as happening in the caprifig.

Dioecy was considered as an adaptation to seasonal climates in order to support the *Ficus Blastophaga* symbiosis (Kjellberg et al., 1987). However, Kerdelhué and Rasplus (Kerdelhué and Rasplus, 1996) proposed an alternative evolutionary scenario in which dioecy would have appeared under the selective pressure of non pollinating fig wasps on the mutualism; this would have led to a reduction of ovary layers in the monoecius fig with the development of a higher number of long-styled (and short-styled) flowers in monoecious trees which successively would have evolved towards dioecy, with male and female trees carrying different types of flowers. The higher expression of floral homeotic protein AGAMOUS in breba with respect to main crop may indicate an original role of these fruits (at monoecious stage) for staminate flowers production, as profichi in the caprifig, since this protein is required for normal development of stamens and carpels in the flower. We suggest that the monoecious ancestor of fig had probably male flowers in the brebas for the pollination of main crop. The development of long-styled pistillate flowers (seeds production), loss of staminate flowers in some trees (female) and an increase of short-styled pistillate flowers (gall production) in other trees (male) were the successive steps for the complete evolution towards dioecy. The colonization of temperate environments from tropical ones was probably a consequence of the dioecy more than a cause (Kerdelhué and Rasplus, 1996). The symbiosis between fig and wasps is also under chemical signals used by the figs to attract the wasps into the ostiole (Souza et al., 2015; Mars et al., 2017) as a possible consequence of the evolution towards dioecy in order to make the volatile chemical signals of seed figs (female) identical to those of gall figs (male) to allow pollination (Borges et al., 2008). The monoecious fig in symbiosis with the wasp had two to three types of fruits for the different generations of the wasp to complete the biological cycle. During this evolution towards monoecy, maybe breba inflorescences also had male flowers close to the ostiole to pollinate the main crop to get either seeds for the fig and galls for the wasp. The late main crop maybe acted as overwintering fruit for the wasps as for the mamme in the caprifig and in spring the cycle started again. In an intermediate evolution caprifig fruits were possibly edible, as nowadays may happen for few varieties. After evolution in male and female trees, the fruits became less palatable in the caprifig (offsprings of the wasp) and more palatable and attractive in the edible fig for the production of seeds to be dispersed by animals eating the fruits. In this evolution towards dioecy, the volatile compounds emitted from the male fig were similar to those of the female fig in order to attract the wasps both for seeds and offsprings as a perfect mutualism (Soler et al., 2012).

## Conclusions

This two-year study, using biological, morphological, anatomical, and genetic approaches showed the positive effect of caprification on qualitative and quantitative parameters of the main crop. Moreover, X-ray images and sections of both brebas and main crop showed a very similar structure at the first stages of development. However, the brebas (fruit bud) differentiated in two seasons passing the dormancy period whereas the main crop (mixed bud) differentiated and developed in the same season. The mixed bud started to develop undifferentiated inflorescence primordia at the end of the winter (with increasing temperatures) which will differentiate almost complete inflorescences within 2 to 3 months both for breba and main crop. The main crop completed its development during the season whereas the breba stopped in summer and developed in the successive spring. Molecular analysis identified a set of key genes involved in the flowering process, fruit development and ripening differentially expressed in both varieties and types of fruit (breba and main crop). This behaviour may be related to the mutualistic coevolution with the pollinating wasp in order to have different fruits to host multiple generations of wasps all year round. A relic of the original monoecy of the species when caprifig and edible fig were probably the same thing, with staminate flowers possibly present in brebas for the pollination of the main crop.

## Data Availability Statement

The original contributions presented in the study are publicly available and can be found in NCBI. BioProject PRJNA623468.

## Author Contributions

AG and GF design of the work. IM, PC, AM, RT, DN, CP, AT, RA, WS, and GF carried out the analysis. IM, PC, RA, WS, AG, and GF carried out the interpretation of data. IM, PC, AG, and GF have drafted the work.

## Conflict of Interest

RA and WS were employed by the company *Sequentia Biotech SL*.

The remaining authors declare that the research was conducted in the absence of any commercial or financial relationships that could be construed as a potential conflict of interest.
