# Supplementary material for: Fruit Development in Ficus carica L.: Morphological and Genetic Approaches to Fig Buds for an Evolution From Monoecy Toward Dioecy
Source: Front Plant Sci. 2020 Aug 21;11:1208. doi: 10.3389/fpls.2020.01208 (PMC7472462; doi:10.3389/fpls.2020.01208)
Supplement: Supplementary file 1 [file DataSheet_1.pdf]

Supplementary material

Fig. S1. Syconium of profichi fruits with sections showing female flowers on the top side of the receptacle (white arrow) and male flowers (black arrow) close to the ostiole (a) and details of the female flower (b top) and male flower (b bottom), respectively.

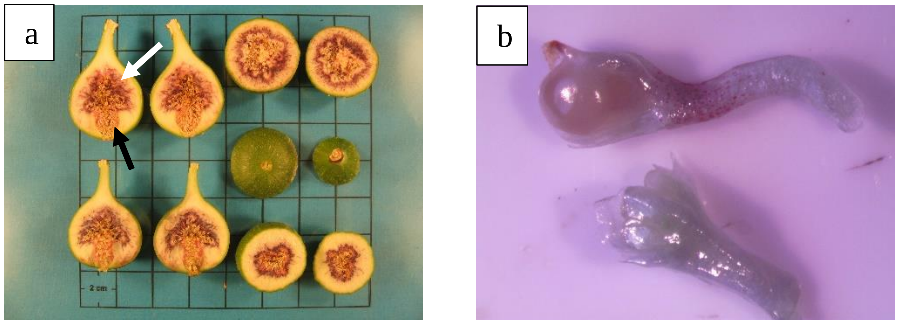

Fig. S2. Two female flowers, the long-styled pistil of the main crop fruits of the edible fig (a) and the short-styled pistil of the fruits (profichi) of the caprifig (b).

a b

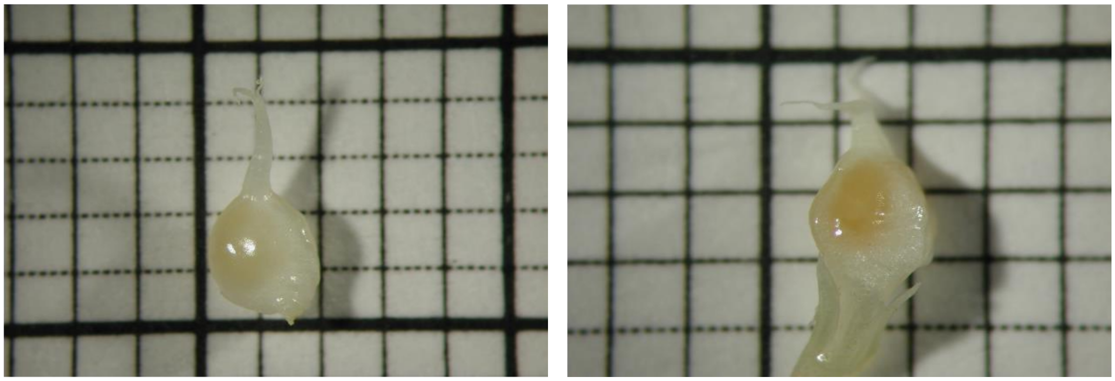

Fig. S3 One-year shoot of fig with the different buds.

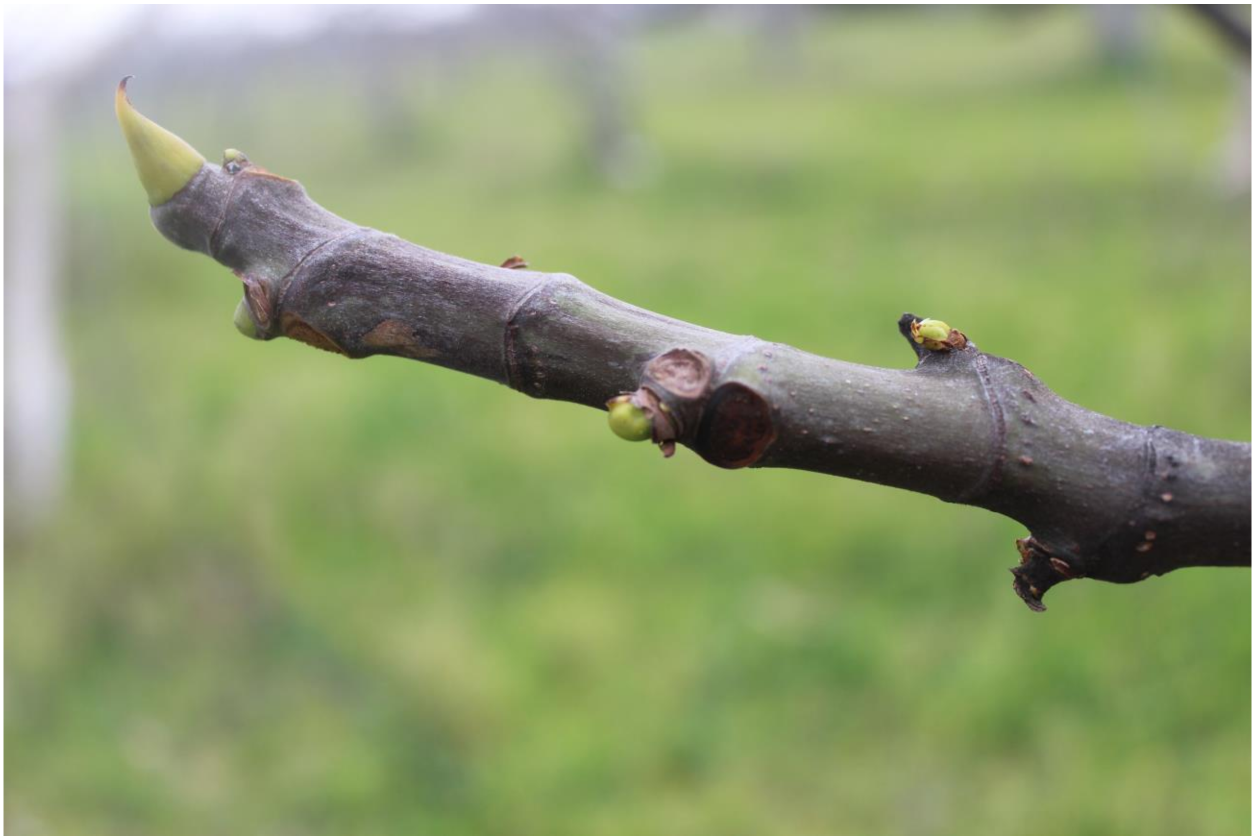

Fig. S4 Bioinformatics pipeline to complete the annotation of the *Ficus carica* L. genome.

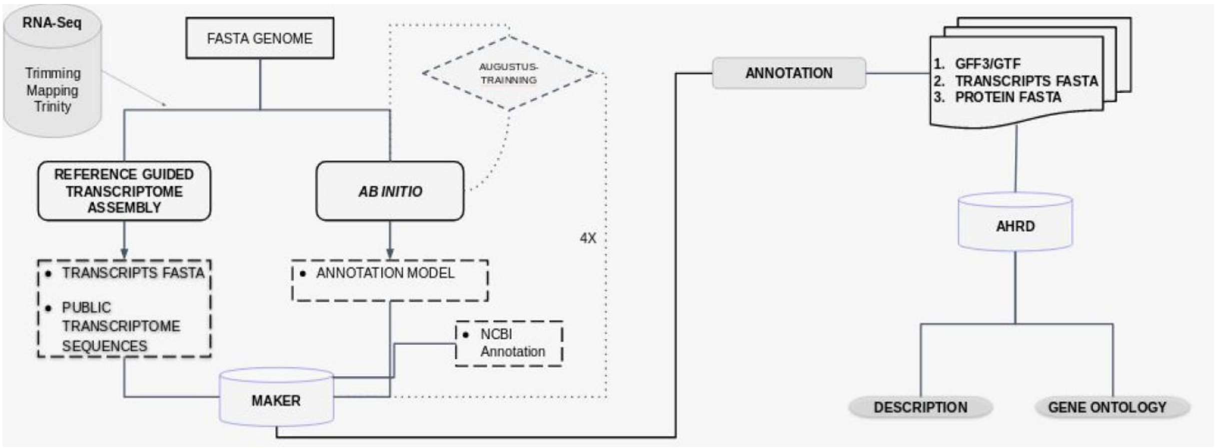

Fig. S5 Shoots of the fig covered with a net (a) to prevent the open pollination from the *Blastophaga psenes*. All the shoot, including leaves and fruits, is covered with the net (b).

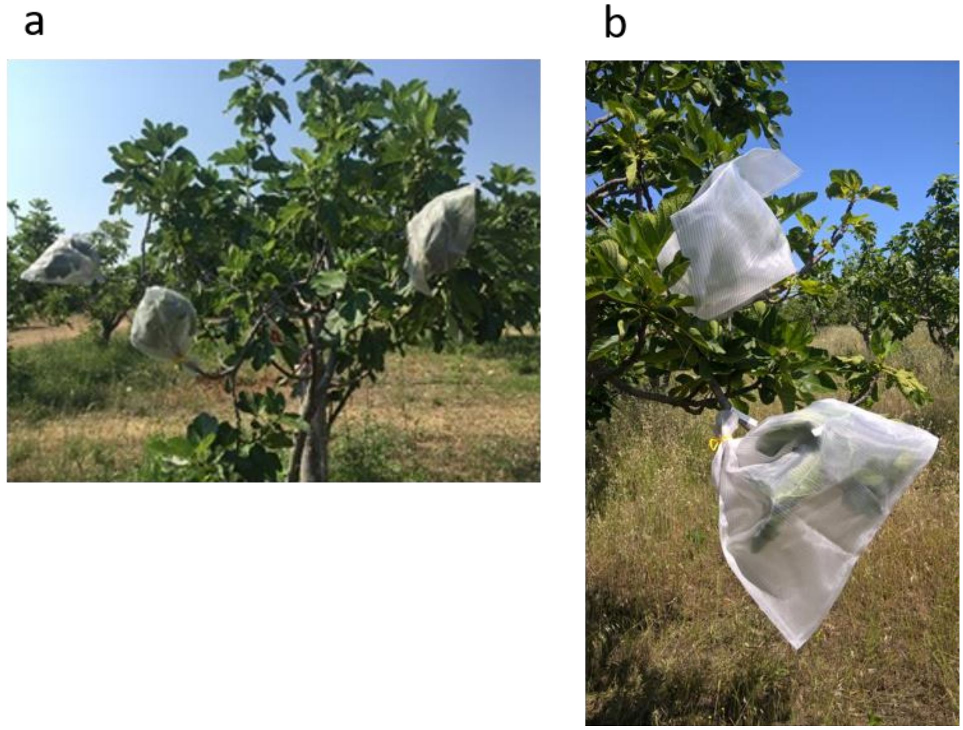

Fig. S6 Hand pollination (a) of the main crop by using a syringe filled with a solution containing pollen grains from profichi fruits of different caprifig varieties. Injection of the solution in the receptive ostiole (b).

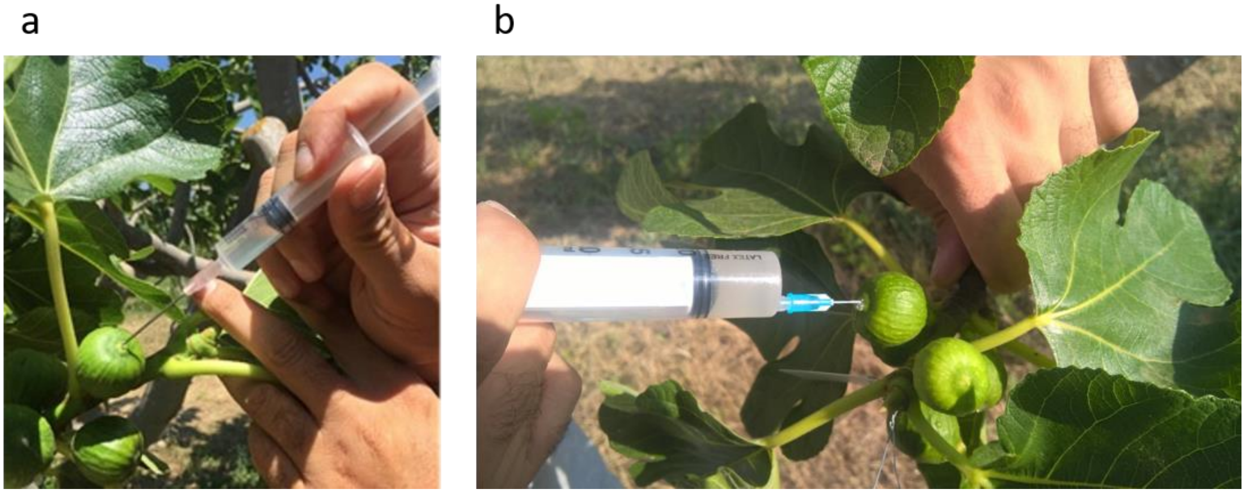

Fig. S7 Graphical representation of the results of the BUSCO.

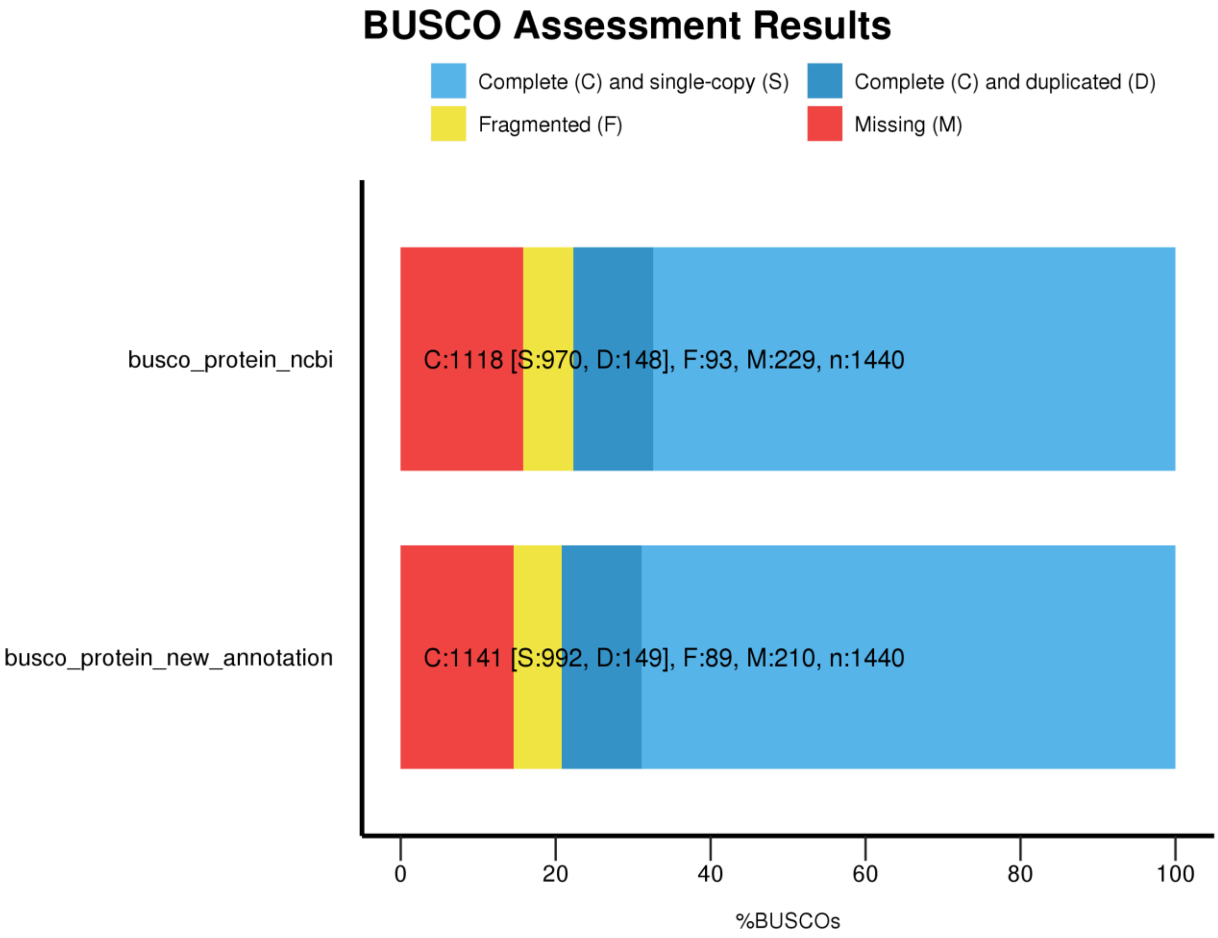

Fig. S8. Number of expressed genes in the two different varieties, Dottato and Petrelli and in the two fruits (breba and main crop) among the 35,567 total genes.

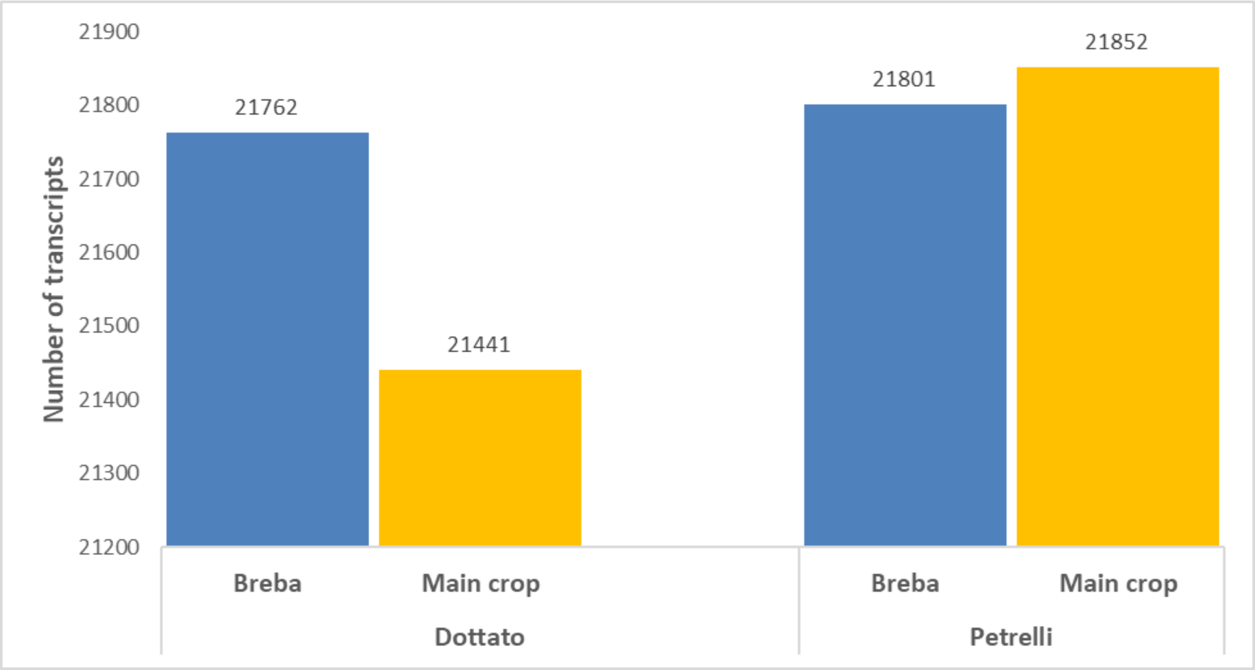

Fig. S9. Venn diagram of the number of the differentially expressed genes between the two varieties Dottato and Petrelli for breba and main crop (a), and the ones expressed only in Dottato or Petrelli for the two crops (breba and main crop) (b). The last diagram (c) represents the number of loci differentially expressed in Dottato and Petrelli in breba and main crop without the one expressed in both fruits.

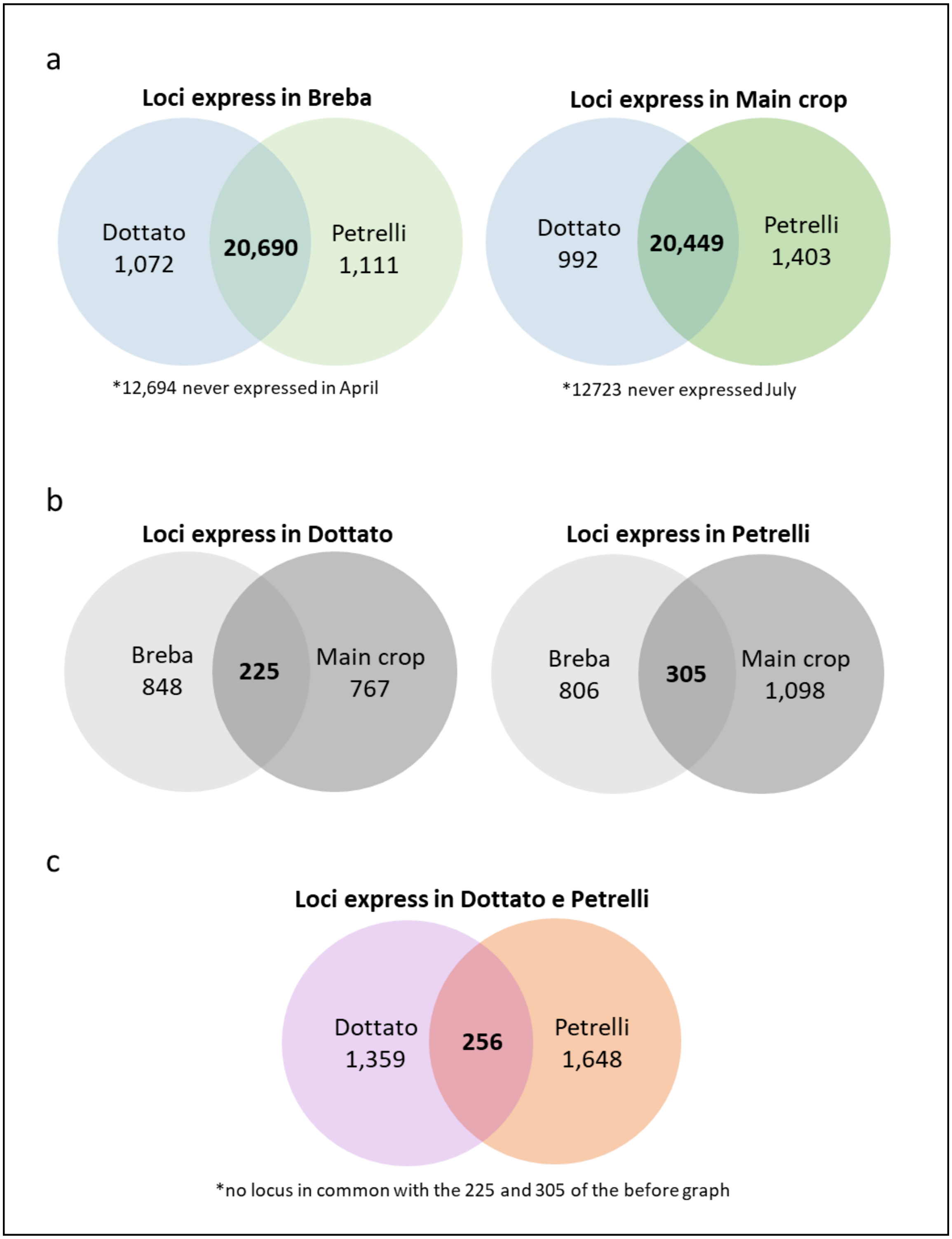

Fig. S10. Analysis of genes, characterized by GO, differentially expressed comparing Petrelli vs Dottato. The number of up-regulated (a) and down-regulated (b) (light-blue bars) was reported together with the expression level (orange bars), grouped by biological category.

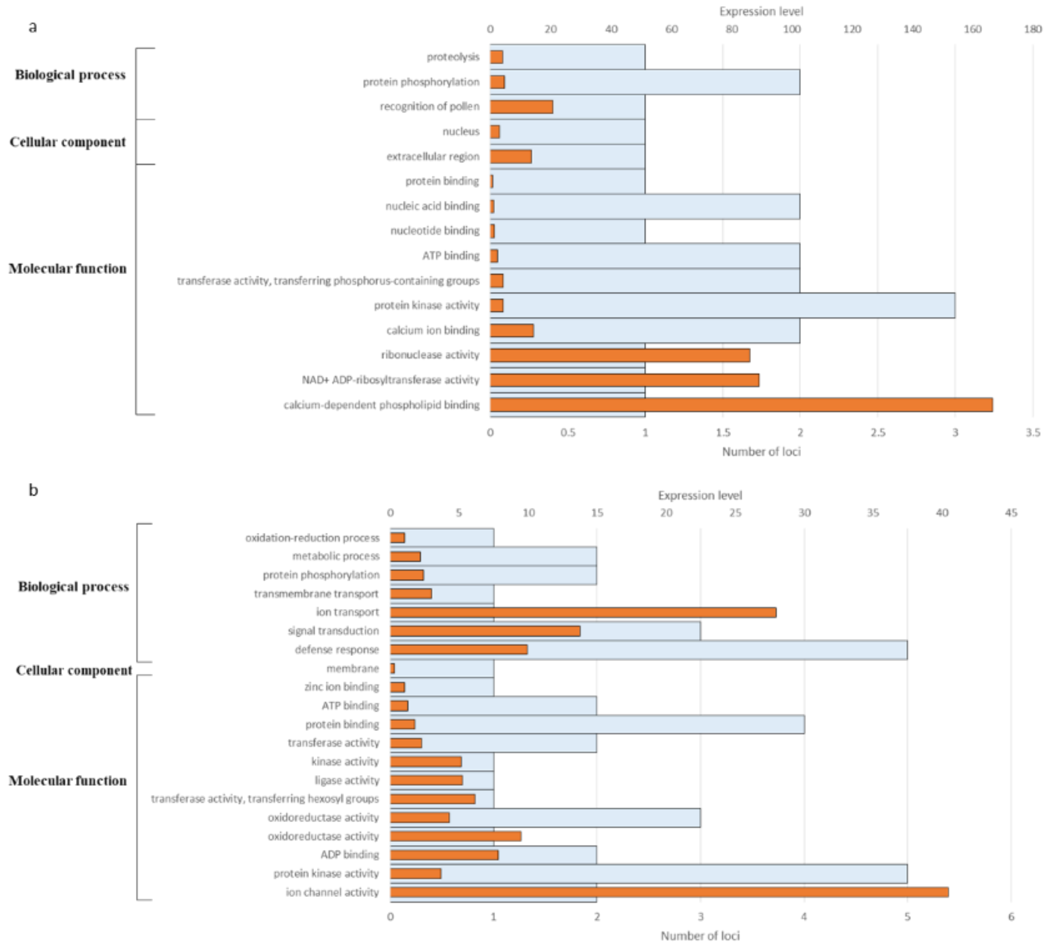

Fig. S11. Fruits of Dottato: hand pollinated, open pollinated and not pollinated (parthenocarpic).

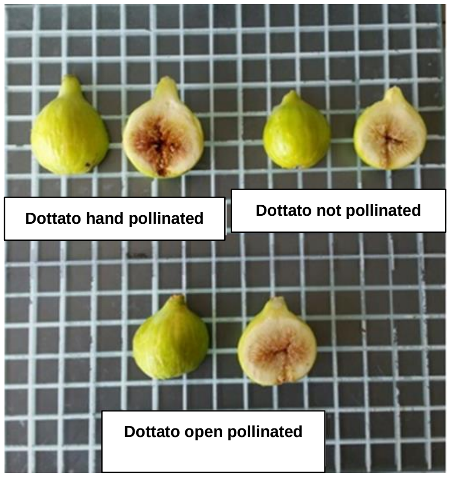

Fig. S12. Fruits of Dottato (brebas) in December (a), February (b), March (c), April (d), May (e) and June (f).

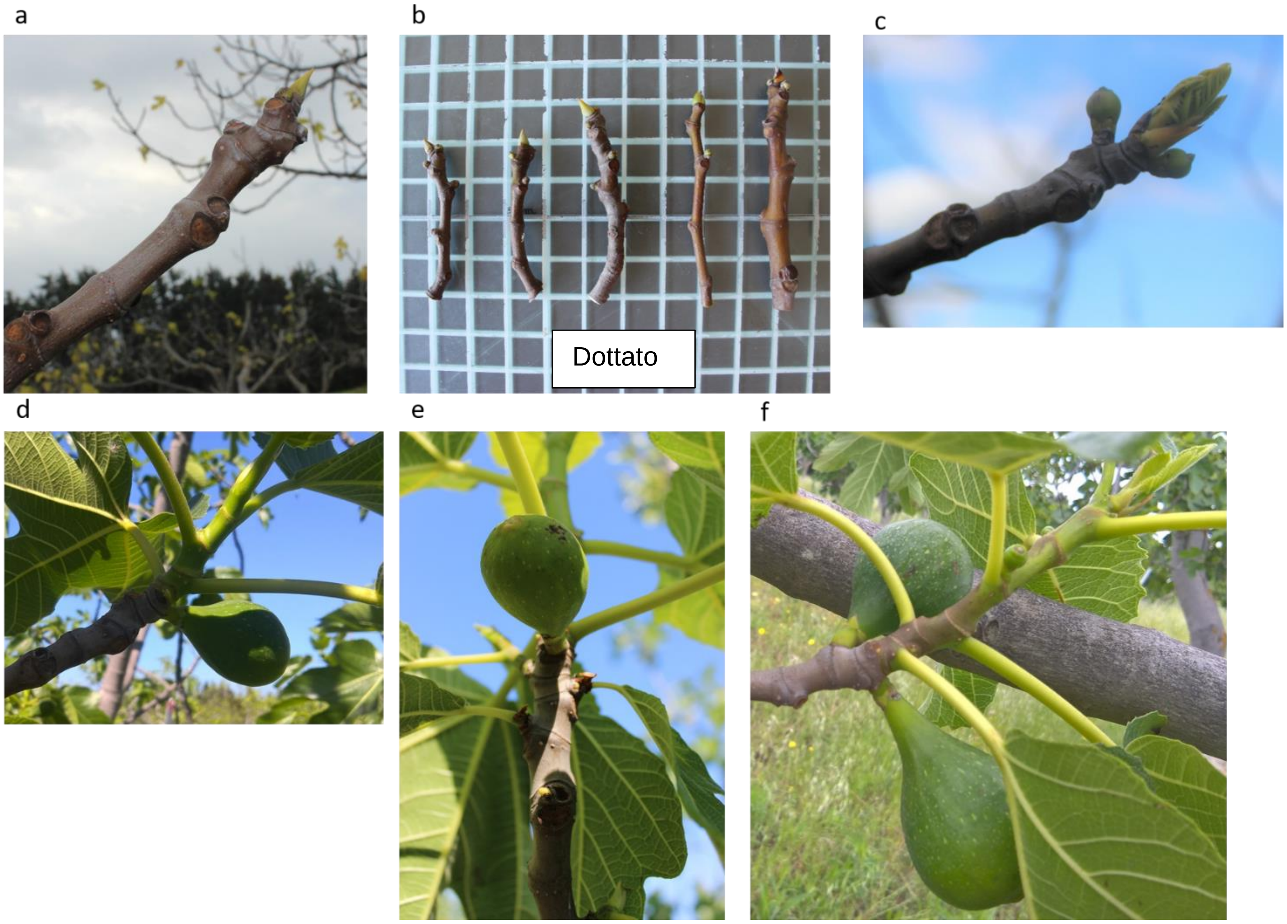

Fig. S13. Fruits of Petrelli (brebas) in December (a), February (b), March (c), April (d), May (e) and June (f).

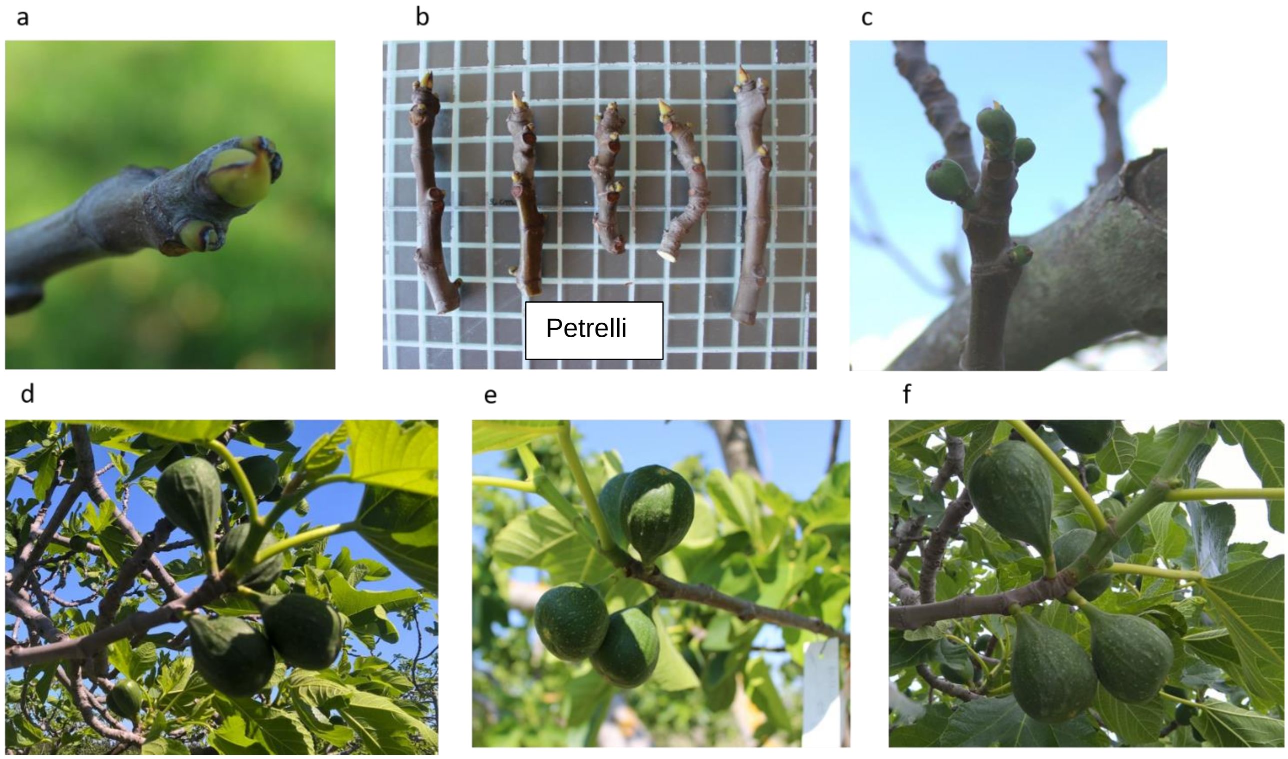

Fig. S14 Sections of bud development visualized by microscopic analyses conducted on fruit (a) and mixed buds (b) of Dottato from July to February.

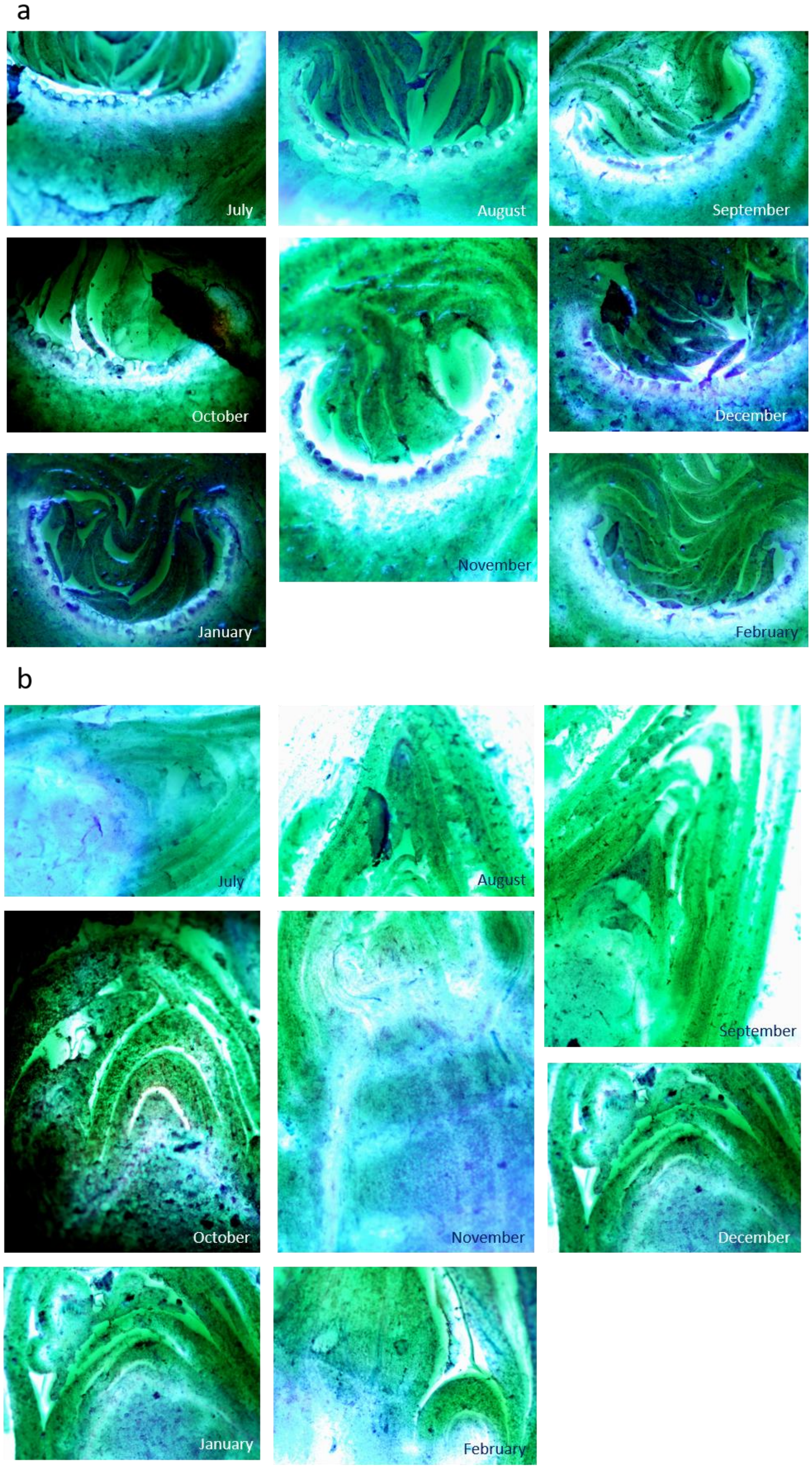

Fig. S15 Sections of bud development visualized by microscopic analyses conducted on fruit (a) and mixed buds (b) of Petrelli from July to February.

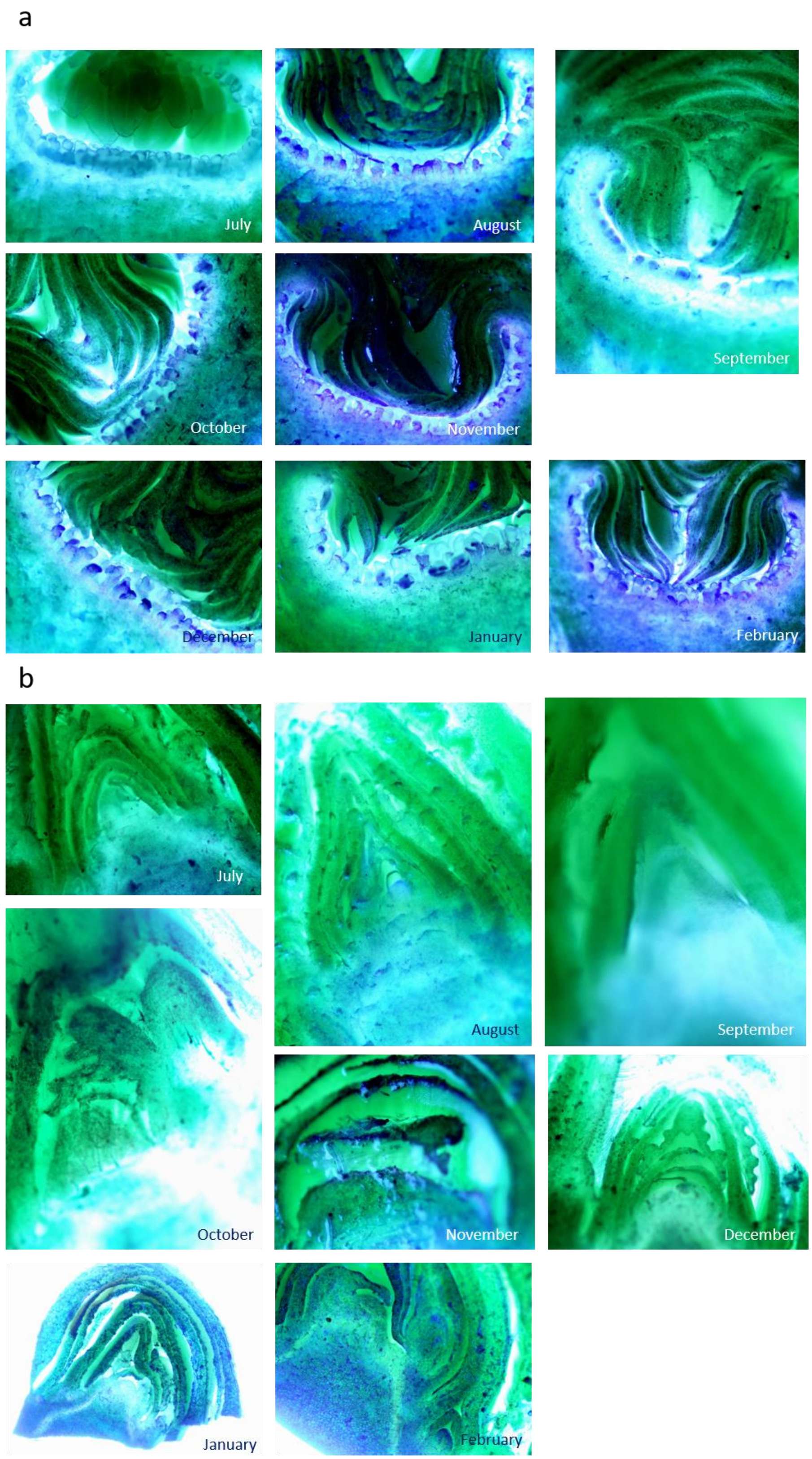

Table S1 List of the 151 loci detected and associated with genes for either flowering or hormones for plant growth, 5 of them identified in *Ficus* genome and 146 through *Morus* database (DB, Dottato breba, DM, Dottato main crop, PB, Petrelli breba, PM, Petrelli main crop).

Gene	Enzyme	Source		*Ficus* Locus	Expression level				Differential expression
		Species	Accession number		DB	PB	DM	PM	
		Ficus carica							
FcFT	flowering locus T		AB594722.1	s01389g28557	12.1	1.5	2.1	30.3	-
ACO2	1-aminocyclopropane-1-carboxylate oxidase 2		KP892660.1	s00021g02797	0.1	0.1	0.0	0.3	-
ACS4	1-aminocyclopropane-1-carboxylate synthase 4		KP892659.1	s00001g00020	3.0	2.3	3.1	8.1	-
ACO3	1-aminocyclopropane-1-carboxylate oxidase-like 3		KP892661.1	s00803g24021	11.3	3.4	34.6	18.8	-
ACS1L	1-aminocyclopropane-1-carboxylate synthase-like 1L		KP892658.1	s00311g15561	0.4	0.0	0.4	3.7	-
		Morus notabilis							
AGL11	Agamous-like MADS-box protein AGL11		EXB88208.1	s00001g00121	226.9	219.7	100.6	63.8	up
AGL15	Agamous-like MADS-box protein AGL15		EXB95305.1	s00133g09774	12.4	8.9	8.4	3.9	up
AGL16	Agamous-like MADS-box protein AGL16		EXB25249.1	s00013g01969	77.4	28.4	36.0	5.6	-
AGL8	Agamous-like MADS-box protein AGL8-like protein		EXB89127.1	s00139g10016	14.7	4.1	47.0	43.9	down
			EXB77653.1	s13459g33826	69.9	32.4	28.2	15.5	-
			EXB77653.1	s07606g32461	120.0	92.4	41.3	35.9	up
AGL9	Agamous-like MADS-box protein AGL9-like protein		EXB40312.1	s00366g16920	65.6	80.9	41.4	68.7	-
			EXB40312.1	s00087g07583	412.1	344.7	150.7	255.0	up
AP2/ERF	Apetala 2-like ethylene-responsive transcription factor		EXC04357.1	s00751g23414	14.1	21.1	24.3	34.1	down
	AP2-like ethylene-responsive transcription factor AIL5		EXB55402.1	s00356g16628	3.7	3.5	1.7	0.3	up
	AP2-like ethylene-responsive transcription factor AIL6		EXC35475.1	s00007g01250	7.8	4.1	1.0	3.5	-
	AP2-like ethylene-responsive transcription factor ANT		EXC12612.1	s00010g01592	26.9	17.7	5.3	2.1	up
			EXC12612.1	s00010g01593	42.3	31.9	8.7	3.3	up
	Auxin efflux carrier component 1		EXB69090.1	s00508g19841	82.1	71.8	24.0	16.9	up
	Auxin efflux carrier component 3		EXB89392.1	s00179g11587	12.5	11.7	19.7	17.0	down
	Auxin response factor 18		EXC48061.1	s00737g23242	8.5	8.6	3.0	2.1	up
			EXB83883.1	s00171g11335	39.6	29.9	23.9	17.2	-
			EXC16214.1	s00280g14722	66.5	54.8	19.1	15.2	up
	Auxin response factor 19		EXB38182.1	augustus_masked-BDEM01000883.1-processed-gene-0.4	92.7	66.8	38.0	38.6	-
	Auxin response factor 2		EXB76510.1	augustus_masked-BDEM01001970.1-processed-gene-0.1	74.4	58.3	47.5	53.4	-
			EXB76510.1	s03769g31377	117.6	71.3	98.1	83.5	-
	Auxin response factor 24		EXB81210.1	s00234g13442	41.8	50.0	64.6	82.5	-

Auxin response factor 3	EXC30555.1	s00158g10824	52.4	34.3	31.4	19.5	-
Auxin response factor 4	EXB98559.1	s00642g22001	51.1	29.2	47.8	19.6	-
Auxin response factor 5	EXC12830.1	s00140g10046	24.4	19.7	31.5	33.9	down
Auxin response factor 6	EXC08253.1	s00231g13375	208.1	164.6	134.5	141.6	-
	EXB39505.1	s00495g19578	290.1	256.6	253.9	227.5	-
Auxin transport protein BIG	EXB77644.1	s00937g25472	31.9	24.5	30.0	35.1	-
Auxin-binding protein ABP19a	EXB38936.1	s00298g15180	1.6	0.5	0.0	0.0	up
	EXB38936.1	s00298g15181	1.8	1.6	0.6	0.1	up
	EXB23832.1	s00014g02106	87.6	199.1	5.5	2.1	up
Auxin-binding protein T85	EXB39313.1	s00065g06285	11.7	8.6	14.0	15.4	-
Auxin-induced in root cultures protein 12	EXB84502.1	s17490g34586	114.4	73.6	101.3	64.3	-
	EXB84502.1	s00079g07154	152.3	131.5	131.9	86.1	-
Auxin-induced protein 5NG4	EXB44370.1	s01296g28050	0.1	0.1	1.7	61.2	down
	EXB82637.1	s00138g09968	1.1	0.6	5.0	7.3	down
	EXB82634.1	s00138g09963	1.6	1.1	3.8	31.7	down
	EXB44371.1	s01296g28049	4.7	14.2	8.7	65.3	-
	EXC31601.1	s00614g21595	6.4	3.8	12.8	242.2	down
	EXC30687.1	s01268g27931	8.4	7.6	7.5	133.6	-
	EXB62699.1	s00262g14235	13.7	2.7	2.3	8.1	-
	EXC13630.1	s00030g03617	14.0	12.2	11.8	11.6	-
	EXC31601.1	s22110g35343	16.6	11.3	11.2	14.6	-
	EXC04202.1	s01814g30025	18.3	14.8	18.9	19.0	-
	EXC17367.1	s00742g23303	20.1	11.1	10.4	6.9	-
	EXC31601.1	s00614g21592	23.9	27.9	21.1	25.6	-
	EXB82636.1	s00138g09967	24.8	22.8	25.9	31.1	-
	EXC31164.1	s00106g08505	30.4	27.1	7.1	9.4	up
	EXB67895.1	s00105g08438	37.0	13.6	7.3	10.4	up
	EXC04202.1	s00151g10568	60.9	57.2	42.0	113.7	-
	EXC25201.1	s00151g10568	60.9	57.2	42.0	113.7	-
	EXC31598.1	s00614g21598	62.7	44.1	125.5	291.0	down
	EXB94908.1	s00029g03514	99.3	224.7	38.7	31.5	-
Auxin-induced protein AUX22	EXC25824.1	s09143g32864	69.2	185.4	9.3	67.3	-
Auxin-responsive protein IAA11	EXB46013.1	s00313g15620	47.0	49.6	32.3	35.2	-
Auxin-responsive protein IAA13	EXC04075.1	s00523g20110	36.0	27.8	18.2	7.5	up
Auxin-responsive protein IAA16	EXB93235.1	s00069g06536	262.8	325.1	198.2	108.4	-
Auxin-responsive protein IAA26	EXB74586.1	s00832g24341	78.1	51.6	36.1	40.9	-
Auxin-responsive protein IAA27	EXB74542.1	s00055g05575	166.8	160.1	96.6	37.2	up
	EXC15862.1	s00053g05391	647.2	510.7	766.9	516.9	-
Auxin-responsive protein IAA3	EXB93236.1	s00069g06534	48.2	62.9	47.3	10.2	-
Auxin-responsive protein IAA32	EXB89357.1	s00081g07271	2.9	8.7	0.8	0.9	up
Auxin-responsive protein IAA33	EXB54743.1	s00296g15142	1.3	3.8	1.1	0.9	-
Auxin-responsive protein IAA4	EXB29840.1	s00275g14570	4.2	3.8	6.1	3.9	-

Auxin-responsive protein IAA7	EXB29843.1	s00275g14569	19.7	20.3	7.5	2.3	-
Auxin-responsive protein IAA9	EXC03899.1	s00010g01653	224.4	237.5	129.7	114.2	up
	EXC01157.1	s00212g12764	703.2	877.2	365.0	264.3	up
ETHYLENE INSENSITIVE 3-like 3 protein	EXB48398.1	s00042g04618	20.1	17.4	15.6	20.3	-
Ethylene receptor	EXB36967.1	s00166g11123	152.0	194.6	101.0	117.2	-
Ethylene receptor 2	EXB98165.1	s00160g10927	57.5	48.2	45.4	83.3	-
Ethylene response sensor 1	EXB95735.1	s00029g03598	78.2	52.8	98.2	165.6	-
Ethylene-insensitive protein 2	EXC16205.1	s00280g14713	60.2	54.3	57.3	61.7	-
Ethylene-overproduction protein 1	EXB54265.1	s01695g29704	31.6	27.1	34.2	51.7	down
	EXB37965.1	s00121g09245	38.3	42.5	38.6	25.9	-
Ethylene-responsive transcription factor	EXB81200.1	s00228g13270	1.5	0.6	1.6	9.4	down
	EXB67204.1	s00034g04000	1.7	4.2	0.3	0.2	up
	EXC01124.1	s00104g08408	4.8	7.3	1.5	13.9	-
	EXB56492.1	s00020g02751	6.7	3.7	8.7	28.0	down
	EXB66082.1	s00023g03057	9.7	5.6	10.6	10.1	-
	EXC17342.1	s00315g15643	10.4	20.8	0.0	0.0	-
	EXC33219.1	s00169g11250	12.6	11.6	7.2	9.7	-
	EXB56928.1	s00012g01806	14.4	7.4	19.5	13.5	-
	EXB38642.1	s00012g01863	20.3	16.7	14.6	9.0	-
	EXB58672.1	s00140g10072	20.6	22.9	20.0	24.9	-
	EXB76737.1	s00589g21182	24.6	62.2	0.0	0.0	-
	EXB54172.1	s00433g18358	28.1	32.7	37.4	31.1	-
	EXB57231.1	s00185g11870	31.5	31.2	52.5	232.1	down
	EXB97053.1	s00601g21390	33.8	30.8	32.0	23.8	-
	EXB83885.1	s00171g11330	48.2	49.5	27.9	4.4	-
	EXB37602.1	s00024g03123	64.8	53.0	131.6	43.2	-
	EXB93691.1	s00011g01745	79.0	97.6	82.7	149.6	-
	EXB38256.1	s00511g19887	585.2	800.6	634.6	1125.4	-
Ethylene-responsive transcription factor 12	EXC30582.1	s00419g18069	19.9	7.7	8.1	4.5	-
Ethylene-responsive transcription factor 1B	EXB54035.1	s01804g30004	2.1	2.2	3.5	21.4	-
Ethylene-responsive transcription factor 2	EXC21056.1	s00185g11867	0.8	1.0	0.2	0.5	-
	EXC02053.1	s00101g08292	75.4	80.9	11.0	18.0	up
Ethylene-responsive transcription factor 3	EXB81318.1	s00002g00368	0.0	0.0	0.2	0.0	-
	EXC25518.1	s00009g01470	59.1	75.9	56.1	107.5	-
Ethylene-responsive transcription factor 4	EXB44623.1	s00317g15702	123.5	161.8	35.1	73.6	-

	EXC30579.1	s00372g17052	145.3	171.3	52.7	60.0	-
Ethylene-responsive transcription factor 5	EXC02051.1	s00101g08295	3.3	2.4	2.3	15.8	-
	EXC02051.1	s00185g11869	124.5	92.0	66.4	223.8	-
Ethylene-responsive transcription factor 6	EXC54525.1	s00101g08300	53.6	56.8	7.1	27.2	-
	EXC54525.1	s00196g12220	69.4	58.7	27.5	35.8	-
Ethylene-responsive transcription factor CRF2	EXC02952.1	s00023g03014	3.7	3.5	2.0	1.2	-
Ethylene-responsive transcription factor CRF4	EXB72466.1	s00525g20146	0.8	0.8	0.2	0.6	-
	EXC22517.1	s00118g09113	1.2	1.3	1.1	0.7	-
Ethylene-responsive transcription factor SHINE 3	EXB85461.1	s07311g32369	0.6	1.4	0.2	0.8	-
Ethylene-responsive transcription factor TINY	EXB74555.1	s00055g05588	9.2	26.8	5.0	3.8	up
Ethylene-responsive transcription factor WIN1	EXB88200.1	s00001g00112	12.7	12.3	9.9	8.2	-
	EXB97710.1	s00748g23382	10.1	6.9	16.6	17.5	down
Ethylene-responsive transcription factor-like protein	EXB99103.1	s00362g16805	12.2	14.0	14.0	10.7	-
Floral homeotic protein AGAMOUS	EXC21999.1	s00824g24272	113.0	78.6	22.9	31.2	up
	EXC21999.1	s00026g03239	145.3	116.3	43.6	26.9	up
Floral homeotic protein APETALA 1	EXB44879.1	s00016g02309	661.8	823.9	353.3	472.1	-
	EXC24730.1	s00433g18352	118.1	94.2	36.1	46.5	up
	EXB84815.1	s00019g02645	123.5	121.7	54.6	33.5	up
Floral homeotic protein DEFICIENS	EXC35487.1	s00151g10560	0.1	0.1	0.0	0.0	up
	EXB48382.1	s00042g04637	6.0	6.9	1.8	1.1	up
Flowering time control protein FCA	EXC27503.1	augustus_masked-BDEM01000163.1-processed-gene-1.14	43.3	38.0	41.6	58.7	-
Flowering time control protein FPA	EXB23115.1	s00987g25910	12.3	9.4	16.6	20.7	down
Flowering time control protein FPA	EXC35026.1	s00385g17337	12.5	11.9	14.6	15.4	-
Flowering time control protein FY	EXB62656.1	augustus_masked-BDEM01001238.1-processed-gene-0.1	26.8	19.9	23.2	24.5	-
Gibberellin 20 oxidase 1	EXC25841.1	s13539g33842	0.0	0.0	0.0	0.1	-
	EXC32582.1	s00046g04963	0.0	0.0	0.0	0.2	-
	EXC11713.1	s00693g22683	1.5	2.0	0.1	0.4	up
Gibberellin 20 oxidase 1-D	EXC20856.1	s00556g20668	1.7	1.0	0.7	0.1	-

Gibberellin 20 oxidase 2	EXB40943.1	s00329g16009	0.7	0.6	0.0	0.1	up
Gibberellin 20 oxidase 3	EXB75633.1	s00328g15975	12.4	7.9	0.5	0.1	up
Gibberellin 2-beta-dioxygenase	EXC29353.1	s00828g24305	1.1	3.3	0.3	2.6	-
	EXC29353.1	s21798g35308	1.5	2.9	0.7	2.7	-
Gibberellin 2-beta-dioxygenase 1	EXC58248.1	s00478g19250	0.3	0.3	2.9	12.9	-
Gibberellin 2-beta-dioxygenase 2	EXB55989.1	s00497g19622	49.3	10.0	6.0	0.7	up
Gibberellin 2-beta-dioxygenase 8	EXC05108.1	s26132g35897	5.4	6.7	2.1	5.3	-
	EXC33074.1	s01261g27885	12.2	8.2	4.0	17.2	-
Gibberellin 3-beta-dioxygenase 4	EXB81619.1	s00518g20009	6.4	4.5	10.6	31.9	down
	EXB81619.1	s00518g20014	32.6	51.1	63.1	53.3	-
Gibberellin receptor GID1B	EXC34655.1	s00126g09485	5.8	6.3	9.7	25.7	down
Gibberellin receptor GID1C	EXB93886.1	s00625g21759	36.3	30.8	45.3	59.1	down
Indole-3-acetic acid-induced protein ARG2	EXB33514.1	s00030g03684	688.1	564.8	1681.7	1779.2	down
Methylenetetrahydrofolate reductase 2	EXB59936.1	s01802g30000	244.8	299.1	179.8	142.0	-
Protein AUXIN SIGNALING F-BOX 2	EXB62856.1	s00050g05216	89.1	95.9	90.7	97.9	-
Protein ETHYLENE INSENSITIVE 3	EXC07787.1	s00217g12920	92.6	72.4	103.8	109.6	-
	EXC07788.1	s00217g12919	420.9	386.4	394.8	500.8	-
putative auxin efflux carrier component 1c	EXC34965.1	s00188g11942	4.3	3.1	1.0	1.0	up
Putative auxin efflux carrier component 8	EXC29925.1	s00027g03360	11.1	3.1	1.3	2.1	up
